# Super-resolution imaging: when biophysics meets nanophotonics

**DOI:** 10.1515/nanoph-2021-0551

**Published:** 2021-12-15

**Authors:** A. Femius Koenderink, Roman Tsukanov, Jörg Enderlein, Ignacio Izeddin, Valentina Krachmalnicoff

**Affiliations:** Center for Nanophotonics, AMOLF, Science Park 104, 1098 XG Amsterdam, The Netherlands; III. Institute of Physics – Biophysics, Georg August University, Friedrich-Hund-Platz 1,37077 Göttingen, Germany; Cluster of Excellence “Multiscale Bioimaging: from Molecular Machines to Networks of Excitable Cells” (MBExC), Georg August University, 37077 Göttingen, Germany; Institut Langevin - Ondes et Images, ESPCI Paris, Université PSL, CNRS, 1, rue Jussieu, 75005 Paris, France

**Keywords:** fluorescence-lifetime imaging microscopy, local density of states, localization artifacts, metal-induced energy transfer, quantum yield, single-molecule localization microscopy

## Abstract

Probing light–matter interaction at the nanometer scale is one of the most fascinating topics of modern optics. Its importance is underlined by the large span of fields in which such accurate knowledge of light–matter interaction is needed, namely nanophotonics, quantum electrodynamics, atomic physics, biosensing, quantum computing and many more. Increasing innovations in the field of microscopy in the last decade have pushed the ability of observing such phenomena across multiple length scales, from micrometers to nanometers. In bioimaging, the advent of super-resolution single-molecule localization microscopy (SMLM) has opened a completely new perspective for the study and understanding of molecular mechanisms, with unprecedented resolution, which take place inside the cell. Since then, the field of SMLM has been continuously improving, shifting from an initial drive for pushing technological limitations to the acquisition of new knowledge. Interestingly, such developments have become also of great interest for the study of light–matter interaction in nanostructured materials, either dielectric, metallic, or hybrid metallic-dielectric. The purpose of this review is to summarize the recent advances in the field of nanophotonics that have leveraged SMLM, and conversely to show how some concepts commonly used in nanophotonics can benefit the development of new microscopy techniques for biophysics. To this aim, we will first introduce the basic concepts of SMLM and the observables that can be measured. Then, we will link them with their corresponding physical quantities of interest in biophysics and nanophotonics and we will describe state-of-the-art experiments that apply SMLM to nanophotonics. The problem of localization artifacts due to the interaction of the fluorescent emitter with a resonant medium and possible solutions will be also discussed. Then, we will show how the interaction of fluorescent emitters with plasmonic structures can be successfully employed in biology for cell profiling and membrane organization studies. We present an outlook on emerging research directions enabled by the synergy of localization microscopy and nanophotonics.

## 1 Introduction

Nanophotonics is the science of light–matter interaction at the nanometer scale, with the dual goals of controlling the propagation, generation, and detection of light on one hand, and on the other hand of detecting, imaging, and manipulating material degrees of freedom with spatial resolutions down to nanometers [1, 2]. The drivers for this field are manifold. A main driver over the past decades has been the control of classical and quantum information. From the viewpoint of classical data transport, classical microphotonics with fibers and waveguides hits the main roadblock that integration density of microphotonics is poor, and on-chip generation and nonlinear operations with light can hardly be achieved [3]. For this reason, there has been a strong push to miniaturize optical waveguides and resonators. A primary aim of tighter confinement of optical modes is not only to improve the potential for higher integration density of photonic components, but also for increasing light–matter interaction strength by virtue of the stronger electric field per photon [4]. Nowadays, nanophotonic structures for enhanced light–matter interaction are developed for a plethora of applications aside from classical and quantum information processing. These applications range from biosensing and molecular spectroscopy [5, 6], to light-induced chemistry [7, 8], nanophotovoltaics, and solid-state lighting [9].

Developments in the field of nanophotonics have been strongly guided by the stringent requirements for increased light–matter interaction [10, 11] that are set by their applicability for cavity quantum electrodynamics in the solid-state. Quantum information processing schemes using light, for instance, require sources that are guaranteed to emit single photons on demand, and which are furthermore indistinguishable in their properties. The state-of-the-art is to use III–V semiconductors with single quantum dots as emitters that are placed in microcavities of high quality factor *Q* and with mode volumes as small as the diffraction limit of light [12]. These microcavities are realized in micropillar resonators [13] and photonic crystal microcavities [14] with exquisite designs to maximize the so-called Purcell factor or local density of states (LDOS) that determines light–matter interaction. Both of these types of structures use purely dielectric materials, and leverage Bragg diffraction induced by wavelength sized periodicity to generate optical band gaps, which provide protection for carefully engineered line and point “defects” (that act as waveguides and cavities) against leakage to the radiation continuum. Nanoscale geometrical features control the precise electromagnetic confinement properties, such as mode-volume electric field distribution and quality factor. Beyond making bright single photon sources lie the challenges of reaching strong coupling and efficiently connecting many such solid-state emitters together in quantum networks. While most mature in the III–V material platform at cryogenic temperature, there is a very strong push to expand these phenomena to other emitters, such as defect centers in diamond [15] or transition-metal dichalcogenide (TMDC) materials [16] with appealing spin properties, as well as to reach operation regimes that are not restricted to liquid helium temperatures.

In the push for developing structures that exhibit strong light–matter interaction at room temperature, plasmonic structures have emerged as alternatives that are complementary to photonic crystals. Plasmonics uses the resonant oscillation of electrons driven by light in nanoscale structures made from noble metals [17]. Nanoparticles can thus act as resonant scattering objects with very strongly enhanced local fields [18–20]. Compared with microcavities, they trade in quality factor *Q* (storage time for light in units of optical cycles) for confinement. Recently reported [21, 22] self-assembled nanoparticle on mirror structures have allowed to reach mode volumes as small as *λ*^3^/10^6^ and measured Purcell enhancements of the order of 10^2^–10^3^, far in excess of what has been achieved in dielectric microcavities. The bandwidths of such antennas are set by the Ohmic loss of the metal and are invariably in the range *Q* ≤ 10–40. The concomitant 20–100 nm spectral bandwidth is commensurate with spectral bandwidths of common room temperature emitters such as dye molecules and colloidal semiconductor quantum dots.

Such optical modes with deep sub-wavelength confinement at visible wavelengths find many uses beyond controlling single photon emitters. Indeed, one of the founding publications on plasmonics is the seminal paper by Stockman [23] proposing the so-called spaser as a nanometer sized plasmonic version of the laser. In this spirit, several groups have recently reported that plasmon antenna arrays coupled to dense fluorescent media can act as nanolasers that leverage nanoscale electromagnetic confinement in concert with distributed feedback [24]. Lasers are not the only light sources that nanophotonics seeks to improve. Although LED lighting is now commonplace, many challenges remain in the field of solid-state lighting. They particularly pertain to very high power-density and high brightness applications, such as required in the automotive industry and for projectors and displays. While efficient blue LEDs are available, a main challenge is the efficient conversion of blue LED light to white light in phosphors, and particular to reach this conversion at very high power densities (current densities reaching A/mm^2^) with simultaneously low material use. Nanophotonic strategies to enhance blue light absorption, to accelerate phosphor emission, and to steer light to create sources of controlled directionality are an active field of research [9]. Finally, the very same nanophotonic structures that can enhance light emission for quantum sources and LEDs are also an important research topic in molecular sensing and chemistry. From the outset, a main driver for the field of plasmonics has been the realization that extreme near field confinement through plasmonic resonances enables Surface Enhanced Raman Scattering (SERS), amplifying the spectroscopic vibrational fingerprint of molecules by factors of $>10^{6}$ [25]. In recent years, this has led to a revolution in vibrational spectroscopy of molecules sometimes coined “molecular optomechanics” [26], and enabled the controlled realization of atomic scale optical mode confinement. Beyond vibrational spectroscopy, nanophotonics is also entering the field of chemistry [8]. Light in very tight confinement can drive chemical reactions through a plethora of effects that include photochemical, photothermal, photocatalytic, as well as hot-electron-driven processes [7].

This review is motivated by the observation that super-resolution microscopy is instrumental in present day nanophotonics, and that conversely nanophotonics can contribute to super-resolution imaging techniques. Common to all of the developing strategies for harnessing light on the nanoscale is that the photonic modes, and correspondingly the light–matter interaction strength, have spatial structures on deep sub-wavelength length scales, down to the single-digit nanometer scale. Understanding nanophotonics thus immediately requires nanometer resolution for the assembly and for the microscopy of systems. Traditionally this has been the realm of near-field scanning optical microscopy (NSOM), wherein a sharp tip is brought close to a photonic mode to convert some of the evanescent field to a signal on a detector. Due to its mechanical raster scanning nature, this is an exceptionally slow approach that furthermore provides only limited spatial resolution (routine *λ*/10, though *λ*/100 can be achieved at visible or near-infrared wavelengths). Also, the detected signal is tough to interpret in terms of the unperturbed electromagnetic fields of the structure at hand, and is not a metric *per se* of, for instance, light–matter interaction strength. In the context of bio-imaging, the last two decades have seen the emergence of a variety of super-resolution techniques using solely far-field optics. With minimal modifications, these far-field super-resolution techniques can be used to image metallic and dielectric nanostructures. Combined with fluorescence lifetime measurements, the strength of light–matter interactions can be directly measured at the relevant sub-wavelength spatial resolution, with the potential to become a powerful experimental tool in the fields of nanophotonic and plasmonics. Conversely, nanophotonic structures can be efficiently put at the service of biophysical observations due to their ability to create huge electromagnetic-field enhancements over deep sub-wavelength length scales. Electromagnetic field enhancements give access to boosted photon count rates on one hand, and thereby access to increased sensitivity or faster dynamic processes. The former has been elegantly exploited in a recent work in which addressable nanoantennas with cleared hotspots, scaffolded by DNA origami nanostructures, increase the average emission rate of single emitters an average of 89-fold, enabling SM detection with a standard smartphone camera for cheap bioassay applications [27]. On the other hand, the deep sub-wavelength structure of the electromagnetic field provides a means to improve spatial resolution. The discovery that sub-wavelength apertures on a metallic film lead to an enhancement of the transmitted light [28] (a so-called zero-mode waveguide, ZMW), triggered the study of lipid membranes [29, 30] and living cell membranes [31], with a spatial resolution of several tens of nanometers and a temporal resolution of microseconds. Even better performances were later achieved with in-plane antenna arrays. These structures show a 10^4^–10^5^ fluorescence enhancement, confined in a zeptoliter-volume nanogap [32]. Such exciting properties were elegantly exploited to study the dynamic nanoscale organization of mimetic biological membranes and the diffusion dynamics of lipids in the membrane of living cells [33, 34]. Thanks to the use of fluorescence correlation spectroscopy (FCS), fast temporal dynamics (of the order of tens to hundreds of μs resolutions) are accessible with a spatial resolution of 10 nm [35, 36]. Another fascinating life-science application of ZMWs is fast and long-read single-molecule sequencing, where individual polymerase molecules are immobilized in ZMWs allowing for watching base-by-base incorporation into a synthesized DNA strand with single-molecule resolution and sensitivity [37]. Recently, this method was enhanced to be able to detect DNA methylation on a single molecule level [38], which is tremendously important for epigenomics.

One of the pillars for resolving biological structures and structural organization below 10 nm is single-molecule Förster resonance energy transfer (FRET), in which dipole–dipole interactions between a pair of single molecules is monitored. Interestingly, the use of sub-wavelength ZWGs can strongly influence the FRET efficiency [39–41], allowing for the observation of FRET at distances that were previously inaccessible [42] and for relative orientations between molecules for which FRET would otherwise be forbidden [43]. Furthermore, it has very recently been shown that the strong fluorescence enhancement generated by rectangle-shaped aluminum ZWGs makes possible the observation of the autofluorescence of single unlabeled proteins emitting very weakly in the UV region of the spectrum [44]. This opens very interesting perspectives in biophysics, allowing the optical detection of proteins without the requirement of potentially disturbing external fluorescent labeling.

**Figure 1:**
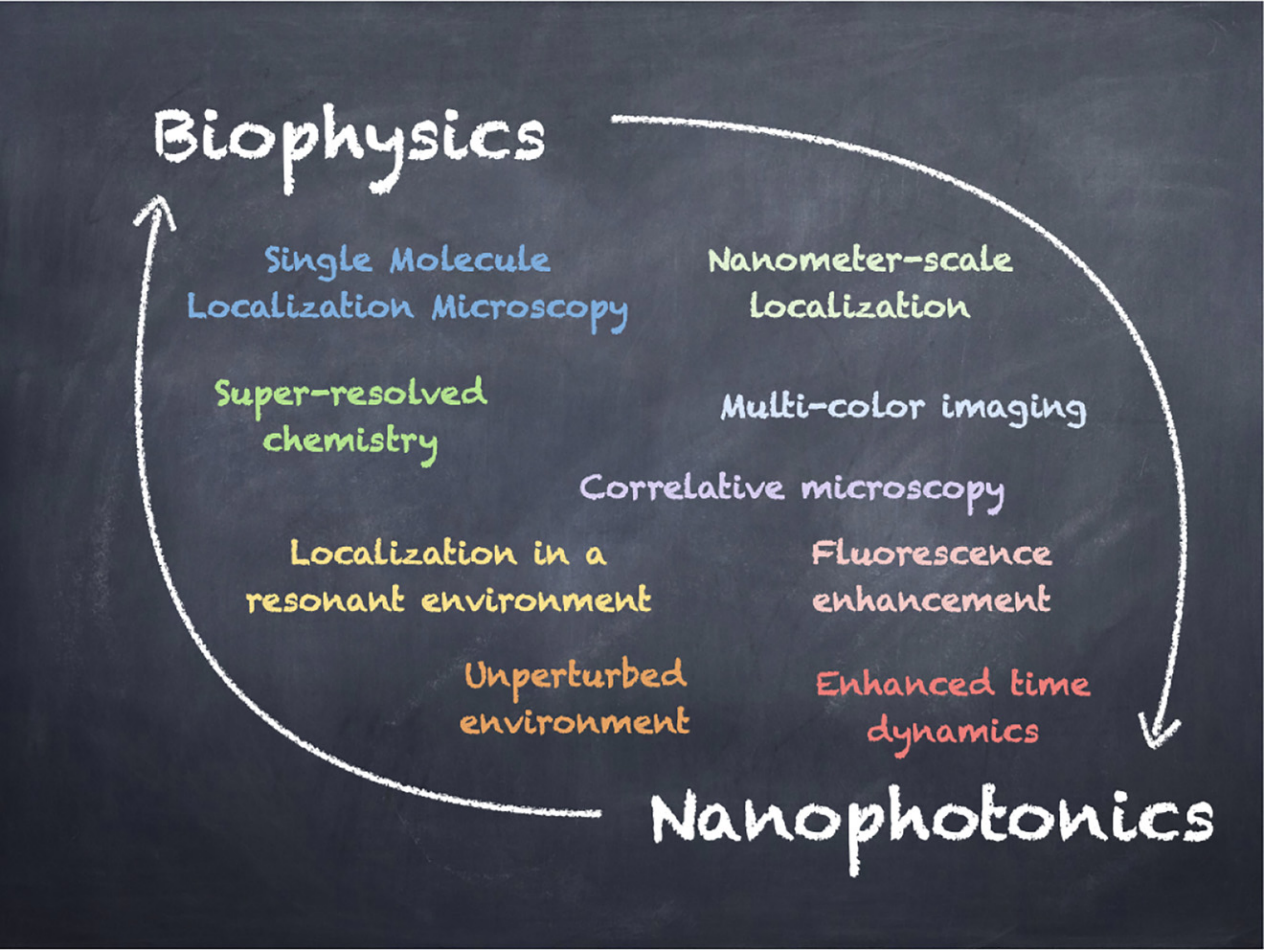
Graphical table-of-content of the review. Interaction between biophysics and nanophotonics can benefit both fields, pushing forward the current technical limits and allowing the study of new phenomena.

This review describes the exciting research at the confluence of nanophotonics and super-resolution imaging in biophysics. As schematically presented in Figure 1, merging the scientific goals and technical developments of both disciplines helps to extend both spatial and temporal resolution, opening new avenues for the development of innovative techniques and for the study of new phenomena. To delineate the scope of this work, this review will be particularly focused on the application of SMLM to the imaging of nanostructured materials, and conversely the use of nanophotonic engineering, particularly of plasmonic structures, to enhance super-resolution imaging of biological samples. This paper is structured as follows: after introducing the basic concepts of single-molecule detection and localization in a homogeneous environment, we will review in Section 2 the principles of the main SMLM techniques commonly used in bioimaging. The most common ways of measuring fluorescence lifetimes will also be detailed. In Section 3, we will introduce all the relevant observables that are accessible when observing fluorescent molecules, such as single emitter brightness, excited state decay rate, radiation pattern, or emission spectrum. These quantities will be linked to the parameters of interest in nanophotonics, such as the local density of states (LDOS) and its radiative and non-radiative contributions. Section 4 reports on state-of-the-art experiments using SMLM techniques for studying the optical properties of nanostructured materials. Both experiments based on measuring fluorescence intensity and measuring fluorescence lifetime are reviewed. A discussion of the main challenges encountered when applying super-resolution microscopy to nanophotonics concludes this part. Section 5 reports several experiments that take advantage of the presence of nanophotonic structures to push further the limits of SMLM, either by extending it to three-dimensional imaging, or by using new generation wide-field detectors capable of fluorescence lifetime measurements. The review then concludes with discussing perspectives of future research directions.

## 2 Far-field super-resolution and lifetime measurements

The advent of super-resolution microscopy [45, 46] has revolutionized optical microscopy over the last ∼30 years, pushing the limits of spatial resolution by two orders of magnitude down to the molecular length scale. E. Betzig, S. W. Hell, and W. E. Moerner were awarded the Nobel Prize in Chemistry 2014 for their achievements in this field, with two separated work principles that are based, on the one hand, on nonlinear techniques and, on the other hand, on super-resolution SMLM with photo-switchable emitters.

The first of these far-field super-resolution methods was STimulated Emission Depletion (STED) microscopy [47, 48], developed by S. W. Hell and co-workers in 2000. STED microscopy uses the intrinsic nonlinearity of stimulated emission in fluorophores to narrow the point spread function of microscopy to the nanometer scale. It was later extended to Ground State Depletion IMaging (GSDIM) [49, 50] and REversible Saturable OpticaL Fluorescence Transitions (RESOLFT) imaging [51, 52]. Similarly, Saturated Structured-Illumination Microscopy (SSIM) [53–56] exploits the nonlinear dependence of the emission rate of fluorophores (optical saturation), in this case excited by a structured illumination pattern.

Conversely, stochastic super-resolution techniques use prior knowledge to beat the Abbe diffraction limit. While the famous Abbe diffraction limit puts a lower bound on the spacing at which two nearby objects can be resolved with an optical microscope, it does not actually constrain the accuracy with which one can pinpoint the location of a single emitter *as long as one has a priori knowledge that the emitter is an isolated single object*. Developments in the field of single-molecule spectroscopy, partly led by W. E. Moerner and co-workers [57, 58], combined with the proposals by E. Betzig to separate the detection of single emitters of densely labeled samples in the far-field, spurred the development of alternative methods that use single-molecule localization in wide-field images [59]. Among these methods are PhotoActivatable Localization Microscopy (PALM) [60], fluorescence PALM (fPALM) [61], Stochastic Optical Reconstruction Microscopy (STORM) [62], direct STORM (dSTORM) [63], Point Accumulation for Imaging in Nanoscale Topography (PAINT) microscopy [64], and more recently MINimal photon FLUXes (MINFLUX) [65], which are all grouped under the umbrella of the acronym SMLM.

### 2.1 Single-molecule localization microscopy basics

**Figure 2:**
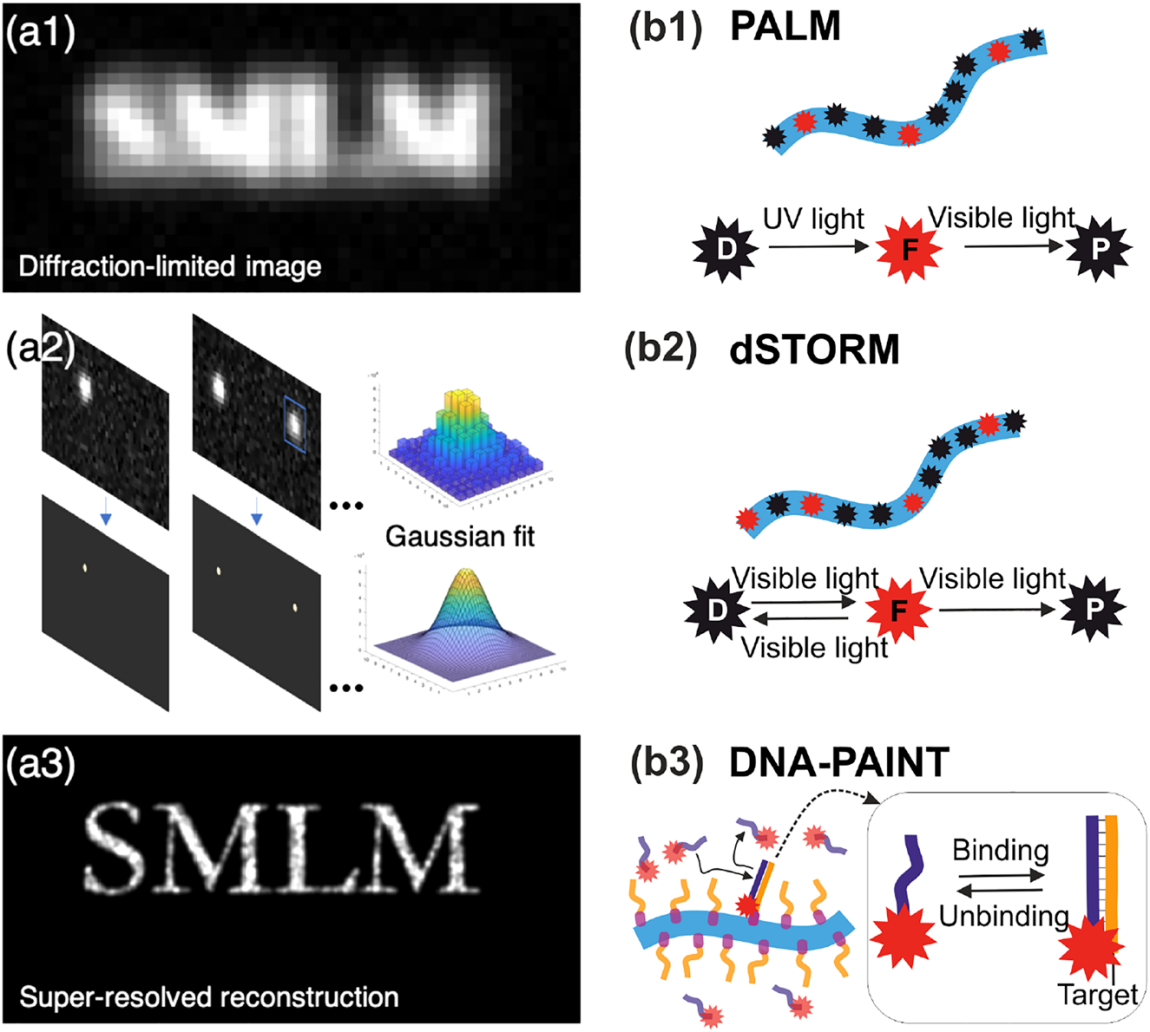
Basics of single-molecule localization microscopy and the most common SMLM-based techniques. (a) Principle of SMLM. In a classical fluorescence microscopy image where all the fluorophores emit simultaneously, the spatial resolution of the image is fundamentally limited by diffraction (a1). In SMLM, single emitters are stochastically activated to become fluorescent, and they are imaged as well-defined individual PSFs until photobleaching. This process is repeated typically for tens of thousands of frames. For each frame, single molecule images are identified and fitted with a Gaussian function to retrieve their center of mass (a2). Subsequently, a super-resolved pointillistic image can be reconstructed (a3), where the chosen size of the reconstruction spot typically reflects the localization precision. (b) SMLM techniques. (b1) In PALM, a fluorophore in dark state D is photoactivated by UV light, it transits into a fluorescent state F, and then undergoes photobleaching (P = photobleached state). (b2) dSTORM uses a stochastic transition of fluorophore between a dark state D and a fluorescent state F achieved by addition of blinking buffer and exposure to the visible light. (b3) DNA-PAINT is based on a transient binding of short piece of DNA carrying a fluorophore, imager strand (violet), against its complementary docking strands (yellow) which are in turn covalently bound to the target of interest, therefore bypassing the limitation of fluorophore photobleaching.

SMLM relies on the fact that one can localize the center position of an isolated emitting molecule with much higher accuracy than the width of the molecule’s image, the latter being defined by the point spread function (PSF) of the system, first determined by Ernst Abbe in 1873 leading to the famous Abbe resolution limit [66] *d*_Abbe_ = *λ*/2NA, where *λ* is the wavelength of light and NA is the numerical aperture of the optical system. Roughly speaking, the localization precision to pinpoint a single isolated emitter scales as the diffraction-limited resolution divided by the square root of the number of detected photons as a direct consequence of the central limit theorem, $\Delta_{loc}\approx d_{Abbe}/\sqrt{N}$, neglecting here for simplicity all sources of noise like background or detector pixelation [67]. For example, a molecule that delivers 10^4^ detectable photons can be localized ca. 100 times better than the classical resolution limit. In a typical SMLM image of biological samples, the localization precision is on the order of Δ_loc_ ≈ 10 nm, depending on the nature of the fluorescent emitter. By recording many images of well-separated molecules by using fluorescent labels that can be switched between non-fluorescent and fluorescent states, one can generate a super-resolved image with a resolution that is in principle limited only by the number of photons detectable from a single molecule and the ability to properly sample the structure of interest (see Figure 2a for an illustration of the principle).

Above, we have stated that the localization precision with which the position of a single emitter can be determined depends solely on the number of detected photons. However, the center of mass of a single-molecule image does not always reflect the true position of the emitter. It is therefore important to introduce the difference between localization precision and localization accuracy. Accuracy refers to the ability to estimate a given parameter, in our case the position of a single fluorescent molecule. Precision refers to the ability to obtain close estimations of the same parameter in a repeated series of measurements. In other words, one can obtain a high localization precision in the estimation of the emitter’s position without reflecting the true position of the emitter, thus having only a low accuracy. This situation of high precision and low accuracy typically reflects systematic errors or statistical biases in the measurements or the chosen PSF model.

The first source of systematic error in SM localization is the assumption that fluorescent emitters act as point-like sources of isotropic emission. Fluorescent molecules are better described as oscillating electric dipoles with an angular distribution of emission that is not isotropic. If the emission dipole is either aligned with or perpendicular to the optical axis of an imaging system, the PSF in the far-field is indeed centered at the position of the dipole. However, in the general case of an arbitrary orientation, the PSF shape will be non-symmetric with a center of mass laterally shifted with respect to the true position of the emitting molecule [68]. Fluorophore orientation can thus be a source of systematic error in its localization, leading to localization errors of up to 100 nm for out-of-focus molecules within the microscope’s depth-of-field [69]. Several approaches, that we will not further describe in this review, can be used to determine the orientation of a fixed emitting dipole, notably including Fourier plane imaging microscopy [70–72], Fourier plane manipulation [73] and filtering [74], or single-molecule polarization microscopy [75, 76].

In bioimaging, we also need to take into consideration that fluorescent molecules serve as tags of other biomolecules of interest. The labeling strategy will therefore play an important role in determining the localization accuracy and precision, and ultimately the final resolution of the reconstructed super-resolution image. On the plus side, the labeling of a molecule of interest with a fluorophore will result in the fluorophore having a certain orientational flexibility depending on the stiffness of the linker between the target molecule and the fluorophore tag. This leads to continuous reorientation of the tag on time scales much shorter than the typical camera integration time. As a consequence, the PSF captured in one frame of the camera in the far-field will be an average of different dipole orientations rendering it symmetric, and its center of mass will accurately reflect the position of the emitter. However, due to the finite linker length, the position of the emitter does not completely coincide with the position of the molecule of interest, and the length of the linker between them will translate into a systematic localization error of the target.

In nanophotonics, the incipient use of SMLM to characterize light–matter interaction in the presence of resonant nanostructures needs also to deal with an additional important source of systematic error due to the efficient coupling between fluorophore emission and nanophotonic structure. The difference between the actual position of an emitter and its estimated position using the center of mass of the far-field emission pattern can lead to mislocalizations of hundreds of nanometers depending on the structure and on the orientation of the emission dipole. We will discuss this problem and outline possible strategies to address it in Section 4.3 and 4.4.

### 2.2 SMLM techniques and labeling strategies

In SMLM, the means to achieve switching of emitters between non-fluorescent and fluorescent states depend on the nature and photophysics of the emitters, as well as the chosen labeling strategy. Here, we will briefly discuss the differences and implications of the main approaches, that are sketched in Figure 2b. PALM uses photoactivatable fluorescent proteins (PA-FPs) that can be one-time photo-converted from a non-fluorescent into a fluorescent state, from where they irreversibly photobleach. When exposed to a photoactivation laser, PA-FPs undergo a conformational change that renders them fluorescent. Since the process of photoactivation is independent for each molecule, the activation of the fluorophores is stochastic, and the number of active fluorophores at a given time must be controlled via the activation laser power. The greatest advantage of this approach is that it is compatible with live-cell imaging, but suffers from a relatively low number of detected photons per fluorophore compared with other methods, having thus a lower localization precision. In the original STORM publication, labeling was done using antibody-based immunostaining with cyanine dyes [62], and the separation in time of fluorescent emission from individual dyes was achieved by inducing stochastic blinking of a Cy5 dye by coupling it together with a Cy3 dye on the same antibody. Shortly after, the development of chemical buffers that induce photoblinking via a redox reaction made it possible to use conventional dyes without the need of a second dye (direct STORM or dSTORM). The high extinction coefficients and excellent photon yields of organic dyes make them excellent candidates to obtain better localization precision compared with fluorescent proteins. dSTORM [63] facilitated the quick spread of SMLM across many biological labs that already had some experience with fluorescence microscopy techniques. While originally the downside of STORM and dSTORM was that, due to the use of antibodies, these techniques were mainly restricted to be used with fixed and permeabilized cells or on the cell membrane surface, the development of innovative labeling strategies and improved chemical dyes has circumvented the need for transfection, allowing imaging in live cells with organic dyes providing better brightness and accessibility to cell biologists [77]. Similarly, the improvement in stability and on/off control of fluorescent proteins for quantitative super-resolution microscopy has blurred the list of pros and cons of choosing fluorescent proteins or chemical dyes for a given experiment.

One of the most recent and potentially most powerful SMLM techniques is Point Accumulation for Imaging in Nanoscale Topography (PAINT) microscopy [64]. In PAINT, a structure of interest is labeled with a non-fluorescent binding target, against which fluorescently labeled ligands can reversibly bind with high specificity. The ligand bound to the target site can be individually seen in a wide-field image and can be localized as in conventional PALM/STORM. The ligand affinity determines the “on-time”, which is the time the molecule is bound to the target site before being released into solution again. The concentration of ligands in solution determines the ligand “off-time”, which is related to the probability with which a ligand finds its target protein. Binding/unbinding events are recorded in a raw data movie and appear similar to blinking events in PALM/STORM, so that the same data analysis procedure as in PALM/STORM can be applied. The core and important advantage of PAINT when compared with PALM/STORM is that PAINT is not limited by photo-bleaching of the used fluorescent dye: any target site is seen again and again each time a new fluorescently labeled ligand binds to it, providing a clear advantage in terms of signal-to-noise ratio. For this reason, PAINT can achieve principally unlimited spatial resolution down to the sub-nanometer length scale.

A special variant of PAINT and the current state-of-the art of this technique is DNA-PAINT [78–80]. Here, the target and ligand molecules are short single-stranded DNA molecules, which thanks to designability of DNA allows for a perfect tuning of binding/unbinding kinetics and which also makes DNA-PAINT highly suitable for multiplexed imaging. The latter can be implemented for fast and simultaneous imaging of multiple targets using kinetic barcoding [81] or, alternatively, imaging of targets one-by-one as realized in Exchange-PAINT [82]. As an example, in ref. [83], different organelles and cellular structures in the same single cell were imaged by using different ligand/target DNA pairs for the different structures of interest, and by performing DNA-PAINT in a sequential manner using a dedicated microfluidic robot that allows to exchange solutions in the sample chamber in a fully automated and highly controlled manner. In the context of using SMLM for nanophotonics, DNA-PAINT is particularly interesting as it allows to repeatedly sample the local environment at the same point-like region where the ligand’s target is functionalized, allowing for a better measurement of light–matter interaction at the nanoscale.

The latest addition to the SMLM family is MINFLUX [65]. The core idea is to scan a sample with a donut-shaped excitation focus, and to deduce a molecule’s position with high accuracy from the recorded photons at various scan positions. The qualitatively new and decisive advance introduced with MINFLUX relies on the fact that single-molecule localization/tracking accuracy increases tremendously by using a donut-shaped focus with zero intensity in the middle instead of a conventional laser focus. With MINFLUX, it is indeed possible to localize single molecules with sub-nanometer accuracy by detecting as few as some hundred photons [65, 84].

Other than the ability of SMLM to pinpoint the position of a point-emitter with nanometer accuracy, which directly depends on the number of detected photons and thus the nature of the fluorophore, another crucial aspect of SMLM is its capacity or lack thereof to properly sample the structure of interest, which directly impacts the final resolution of the pointillistic reconstruction of the sample. An image can be reconstructed with precisely localized molecules, but the labeling density does directly affect the capability to resolve features of interest in a sample. The relation between labeling density and the ability of correctly reconstructing sample features is quantitatively described by the Nyquist–Shannon sampling theorem. In short, in order to claim an image spatial resolution of *X* nm, one needs to sample the structure at a minimum of *X*/2 nm. This means that one needs to factor in both the localization precision and the labeling/sampling density in order to claim a given image spatial resolution. In practice, the resolution does also depend on a multitude of other factors such as systematic biases, the underlying spatial structure, data processing and so on. Today’s standard approach to determine the spatial resolution of a super-resolved image without any *a priori* information is to use Fourier ring correlation (FRC) that allows for assessing the spectral image-content signal-to-noise ratio [85], a technique commonly used in cryogenic electron microscopy (cryo-EM). On that note, the adoption of data analysis algorithms originally developed for cryo-EM have the potential to unravel new information in SMLM experiments. One example can be found in the work of Salas and coworkers, who applied cryo-EM analysis and 3D reconstruction approaches to two-dimensional SMLM data of macromolecular structures [86]. While in this section, we have described the principles of 2D SMLM super-resolution experiments, we will develop further the challenge of axial super-localization of molecules in Section 5.1.

### 2.3 Fluorescence lifetime measurements

Until now, we have discussed how SM detection can be used to retrieve the position of molecules of interest with nanometer accuracy and some of its limitations. In the standard implementation of these techniques, however, the information provided by the fluorescence lifetime is not retrieved, mainly due to the difference in time scales needed to take a SMLM image (tens of milliseconds) and the typical fluorescence decay times on the nanosecond timescale. However, the fluorescence decay time (excited state decay rate) can carry precious information about the local environment of an emitter. For example, the fluorescence lifetime can be exploited to study light–matter interaction in nanophotonics; to probe changes in the local environment like viscosity, pH or polarization; to study conformational changes and dynamics of macromolecules; or to monitor molecular interactions, among many other applications. Fluorescence lifetime imaging microscopy (FLIM) is thus a powerful technique used to distinguish between different emitters and/or to probe the local environment of an emitter.

**Figure 3:**
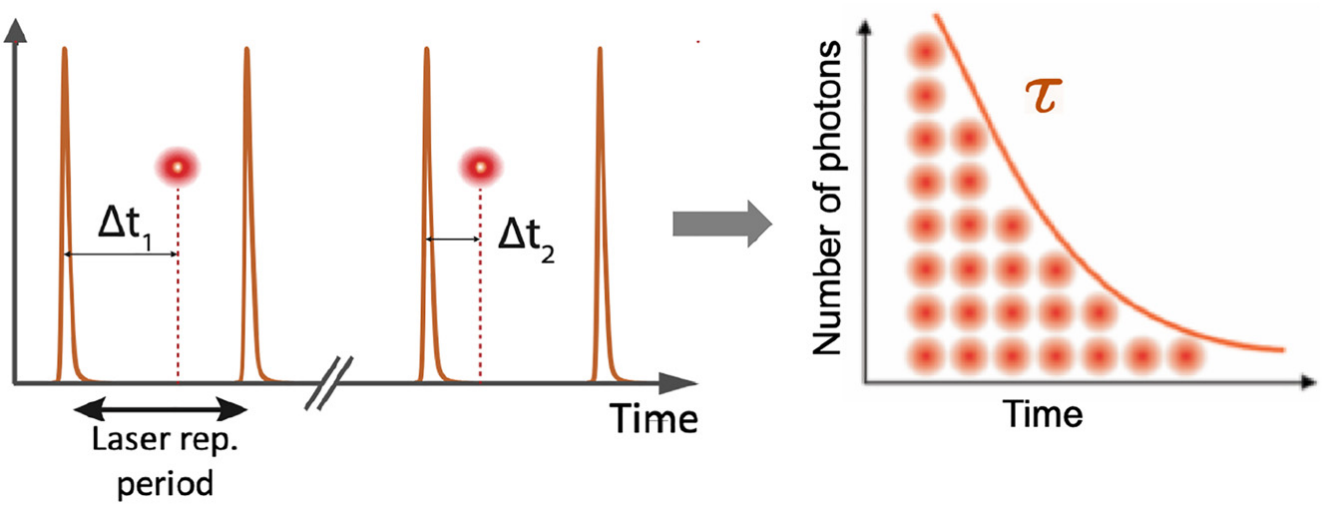
TCSPC principle for fluorescence lifetime measurements. A pulsed laser excites an emitter to its excited state, and the detection times of individual fluorescence photons are repeatedly measured and digitized by the TCSPC electronics timer (left panel). Arrival times Δ*t*_*i*_ are retrieved to build a fluorescence decay histogram over time. An exponential fit of the histogram is typically used to retrieve the characteristic fluorescence decay time *τ* (right panel).

While there exist many different techniques to measure fluorescence lifetimes, time-correlated single-photon counting (TCSPC) is considered the most robust and sensitive, as it is independent of excitation intensity fluctuations and operates at shot noise level (single-photon detection). In the weak-coupling regime, the excited state of an emitter decays exponentially in time with a characteristic decay time, its *fluorescence lifetime* *τ* = *γ*^−1^, where *γ* is the spontaneous decay rate. In TCSPC, the detection times of individual photons after pulsed excitation are digitally measured with an electronic timer. The recorded detection delays Δ*t* are then used to build a discrete histogram of representing the fluorescence decay curve (see Figure 3).

However, the resulting TCSPC histogram of single-photon detection times will represent the actual fluorescence decay only if two strict conditions are observed: maximally one photon can be counted during one excitation/detection cycle and the time between two consecutively detected photons has to be larger than the dead time of the detector and electronics (on the order of a few tens of nanoseconds). This has the effect that at high count rates, when the time between detected photons approaches this dead-time, one does no longer detect all photons hitting the detector, which leads to a distortion of the finally measured fluorescence decay curve (so-called pile up). Therefore, standard TCSPC measurements set the photon excitation/detection rate to ca. 0.01 of the maximum possible detection rate to obtain TCSPC histograms that reflect the true fluorescence decay. Moreover, the time between two subsequent laser excitation pulses should be roughly 5 times longer than the fluorescence decay time to allow for a unique assignment of a detected photon to the correct laser pulse that generated it.

Ideally, the laser excitation pulses as well as the instrument response function (IRF) of detector and electronics should be perfect delta functions, so that the measured TCSPC histogram would be only determined by the fluorescence decay properties. However, in practice, this is never the case. This means that when evaluating TCSPC histograms, one has to take into account the finite temporal width of the excitation pulses and the IRF of the TCSPC measurement system. Both these quantities, pulse width and IRF width, ultimately determine the shortest measurable lifetime. The *precision* with which a given fluorescence lifetime can be estimated does ultimately depend on the number of collected photons (shot noise), but also of additional noise (electronics noise, jitter), and in the case of multi-exponential decays, on the proximity of the different decay times [87].

## 3 Relevant photophysical quantities

The main measurement scenario considered in this review is that of obtaining super-resolved information from fluorescence microscopy data that is obtained from single fluorophores at a time. This information is then used as a reporter of geometry, local physico-chemical environment, and/or local nanophotonic mode properties. In this section, we define the main observables that are accessed by an experimentalist in fluorescence microscopy, and we will link them to nanophotonic parameters that can be measured in super-resolution imaging. Each of these observables is impacted and can be manipulated by the nanophotonic environment of an emitter.

### 3.1 List of observables

**Figure 4:**
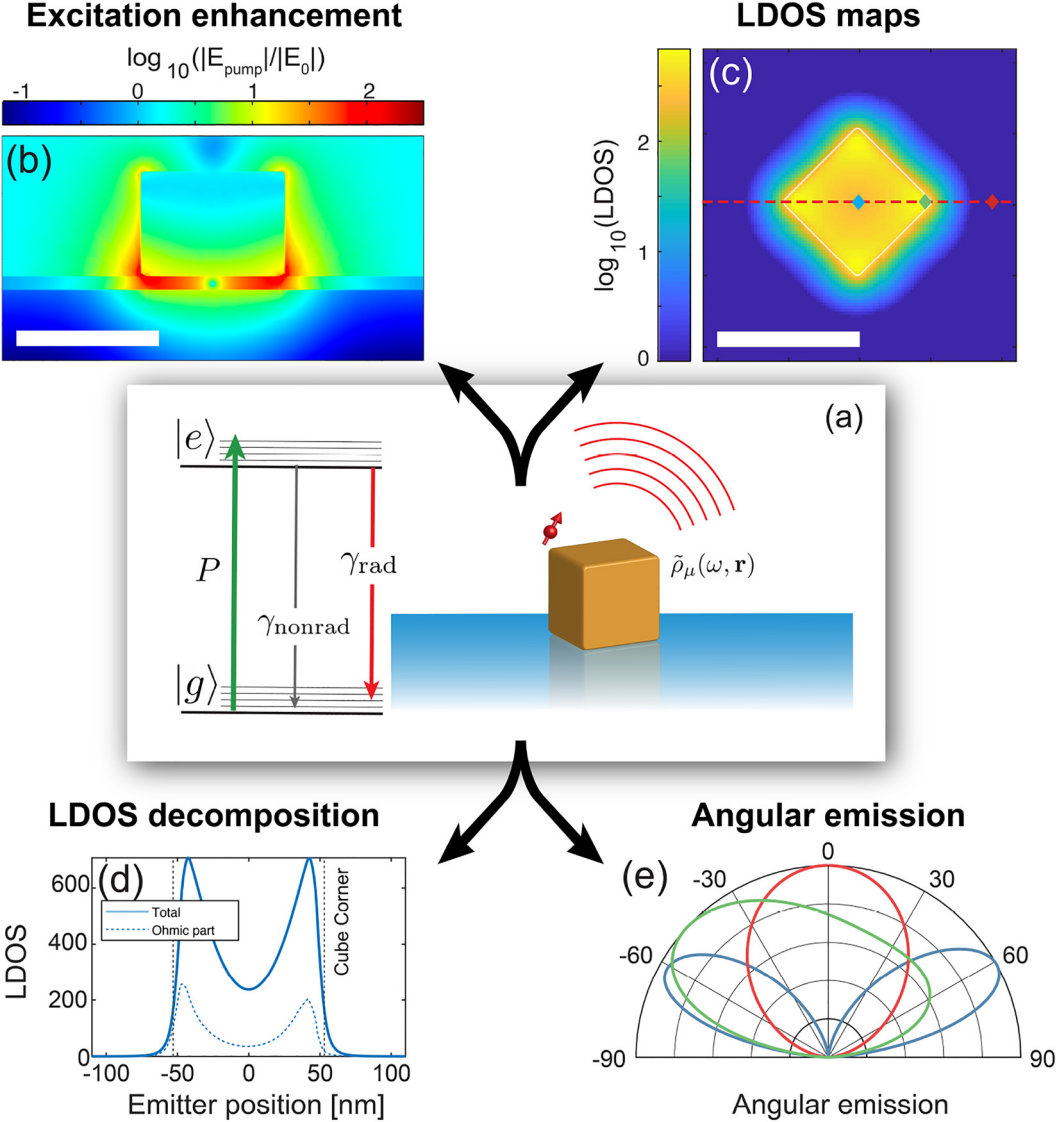
Photophysics of a quantum emitter in the near field of a nanophotonic structure (a) Sketch of solid-state emitter (transition dipole moment µ) next to a nanophotonic resonator, schematized as the cuboid object, to illustrate photophysical and nanophotonic concepts. The emitter is pumped at rate *P* from the ground state |*g*⟩ into the excited state vibrational band |*e*⟩. From there, it can decay radiatively (rate *γ*_rad_), or non-radiatively (intrinsic rate *γ*_nonrad_). The nanophotonic structure can accelerate the rate of decay through the local density of states ${\tilde{\rho }}_{\mu }\left(\omega ,r\right)$ to $\gamma_{\text{nonrad}}+\gamma_{\text{rad}}{\tilde{\rho }}_{\mu }\left(\omega ,r\right)$, where ${\tilde{\rho }}_{\mu }$ may itself fall apart in contributions to free space, loss in the resonator, and/or guided modes. (b–e) Calculations of pump field, LDOS, LDOS decomposition, and radiation patterns for the case of an Au nanocube on an Au mirror, spaced by a 10 nm oxide layer, as pioneered by Akselrod [21]. We assume a 75 nm cube (Lorentz–Drude model for dielectric constant). Panel (b) shows field enhancement for external driving (linear polarization in the plane of plot, 710 nm wavelength). Panel (c) shows an LDOS map in the plane midway the spacer at about 680 nm. The LDOS is strongly dependent on where the source is located under the cube. (d) Cross cuts in LDOS, decomposed in radiative and nonradiative part, for a dipole orientation µ = (2/3, 1/3, 2/3) (arbitrarily chosen), and along the cube diagonal as indicated. There is an almost three orders of magnitude variation over a 100 nm distance. For this frequency (chosen in between two resonances for illustration purposes), the LDOS is due to the superposition of two antenna modes, each with a very different radiation pattern. The radiation patterns in (e) show strong position dependencies and asymmetries, due to the far-field interference of the cube modes (three choice antenna positions indicated lozenges in (c)). Results obtained by COMSOL Multiphysics, using the RETOP package [88].

We consider the standard picture for molecular emitters, sketched in Figure 4a. If a fluorophore is illuminated by a pump laser of intensity *I*_*P*_ [W/m^2^], there is a probability for the molecule to absorb a photon and to become excited from its electronic ground state |*g*⟩ to its electronic excited state |*e*⟩. This probability is set by the molecular absorption cross section *σ*_*a*_ at the pump energy ℏ*ω*_*p*_. The pump rate, i.e. the rate at which the fluorophore goes through excitation–emission cycles, is set by $P=\frac{I_{P}}{\hbar \omega_{p}}\sigma_{a}$. After excitation, the fluorophore rapidly (within picoseconds) thermalizes with the environment, leaving the electron in the lowest vibrational level of the first electronic excited state. From there, usually a set of available decay channels is available, which includes non-radiative decay channels that do not lead to an emitted photon, as well as radiative decay channels to a single, or to various final states. The probability that a given excitation cycle leads to an emitted photon is quantified by the quantum efficiency QE of emission (quantum yield), which can be written as QE = *γ*_rad_/(*γ*_rad_ + *γ*_nonrad_). This highlights the fact that the quantum efficiency is the result of a competition between two rates: *γ*_rad_ is the radiative decay rate connected with fluorescence emission while *γ*_nonrad_ quantifies the intrinsic non-radiative decay rate of the molecule. The total decay rate is the inverse of the fluorescence lifetime. The radiative rate is determined by the fluorophore’s oscillator strength according to Fermi’s Golden Rule. The emitted photons are spectrally red-shifted in energy (Stokes shift), and there is no coherence between emitted photons, or between emitted and absorbed photons. Once a photon is emitted, it is not necessarily detected. Instead, the detection probability is defined by the angular distribution of radiation (radiation pattern) and its overlap with the collection aperture of the optics, and finally by the quantum efficiency of the detector. This picture leads to the following list of observables:

**Brightness/collected photon flux** CCD/CMOS cameras and single photon counters ultimately count the number of photons per time collected from a fluorophore. The collected number of photons per unit time is set by the product

$$\frac{I_{P}}{\hbar \omega_{p}}\sigma_{a}\times QE\times C\times \eta$$

where the collection efficiency *C* is itself an overlap integral of the angular distribution of radiation (property of the sample) and the geometry of light collection (e.g. NA, setup properties), while *η* is the detector efficiency [20, 89, 90].

**Total fluorescence decay rate** Time-correlated single photon counting measures the distribution of arrival times of photons relative to laser excitation pulses. From the resulting decay traces, one can extract the total decay rate *γ* = *γ*_rad_ + *γ*_nonrad_ (inverse fluorescence lifetime) as explained in Section 2.3.

**Quantum efficiency** The quantum efficiency QE or quantum yield of fluorescence is a quantity that is not directly accessible by experiment, as it defines the ratio of total emitted photons (emitted in all directions) to total absorbed photons. Since the pump rate and collection efficiency of photons are highly convoluted quantities, assessing the quantum efficiency of single fluorophores is extremely challenging. An important technique is to compare pulsed and continuous-wave (cw) excitation to explore emitter saturation. When saturating fluorescence with pulsed excitation (pulses much shorter than the fluorescence decay rate, and repetition frequency much slower than fluorescence decay), the pump rate approaches the laser repetition frequency. This nonetheless leaves the photon collection efficiency as an unknown. An excellent review of the difficulties of measuring quantum efficiency is provided by ref. [91]. An elegant absolute and calibration-free technique of measuring quantum yields of single emitters is to measure the modulation of the fluorescence emission rate in the vicinity of a planar metallic interface [92–103].

**Radiation pattern** The radiation pattern (angular distribution of emission) of single nanosources is the distribution of light over the far-field angular degrees of freedom, and can be viewed as the probability per steradian that the emitted photon travels into a given direction. Back-focal plane microscopy, also known as Fourier microscopy, *k*-space microscopy or conoscopy, can be realized with standard high-NA fluorescence microscopes to record the radiation pattern within the light collection cone of a microscope objective [70], [71], [72, 104, 105]. This technique was first applied to single molecules by Lieb et al. [70], demonstrating the strong modification of the radiation pattern of fluorophores close to dielectric interfaces, depending on dipole orientation and emitter-interface distance, as mentioned in Section 2.1. Instead of Fourier imaging, one can also use defocused imaging to reconstruct information about the orientation of emitters [71]. Defocused imaging does not strictly report radiation patterns, as opposed to true Fourier imaging. When going out of focus, the resulting interference pattern of an emitter depends on the relative amplitude and phase of light emitted along different angles, and thereby on the radiation pattern. We note that holography allows phase resolved Fourier plane imaging [105], but is difficult to apply to non-coherent fluorescence emission.

**Spectral properties of emission and excitation** Finally, all the properties outlined above can be measured as functions of excitation and/or emission wavelengths. Excitation spectroscopy of single emitters is highly challenging, but can report on the spectral structure of emitter pump rate, either modulated by absorption resonances in the matter, or by structures in the pump light. In emission, spectral changes as compared with “free emitters” can report on relevant physics in two ways. First, if the overall (4*π*-sr integrated) spectral distribution of emitted light is modified, this indicates a modification of the branching ratio, i.e. the relative likelihood, of competing radiative and nonradiative processes. Second, even if branching ratios are unchanged, the radiation pattern of a source may be modified due to angular and spectral filtering effects. An emitter can thus be used as an “internal light source” to perform spectroscopy on transmission probabilities from inside a sample to a far-field detector.

### 3.2 Nanophotonic parameters of interest for super-resolution imaging

The use of super-resolution microscopy in the domain of nanophotonics has been mainly driven by the desire to precisely map the figures of merit of nanostructured environments for controlling light–matter interaction. We discuss the physics of spontaneous emission control in nanophotonic resonators to explain what the main electrodynamic quantities of interest are. Nanophotonic resonators can enhance the brightness and emission decay rate of fluorescent sources in a number of ways, namely through pump field enhancement, local density of optical states (LDOS) modifications that can accelerate photon emission but also can induce quenching, or emission directivity [18, 20, 89, 90]. These concepts are illustrated in Figure 4b–e for the case of a nanocube-on-mirror system. This interesting nanophotonic resonator was pioneered by Akselrod et al. [21], and consists of a (mono-crystalline) Ag or Au cube deposited on an Au mirror, with just a nanometric spacer. This system shows simultaneously strong pump field enhancement, high spontaneous emission enhancement, and directional far-field emission. Throughout this section we focus purely on the *electromagnetic* aspects of fluorescence control. Particularly in plasmonic systems, and at very small separations between fluorophore and metal, also electronic and chemical effects can occur that can dramatically change emitter properties. Examples are conformational changes of fluorophores in extreme confinement, electron transfer processes between fluorophore and metal, and promotion or inhibition of photochemical modifications of fluorophores as they are optically cycled. For the strict purpose of mapping nanophotonic figures of merit, these are undesired, parasitic effects. Conversely, nanostructure-assisted photochemistry is a topic of large interest in itself, to which we briefly return in the outlook section.

The detected intensity of a fluorophore in a microscope is generally understood to be enhanced by a nanophotonic structure due to the product of three factors (subscript “ref” to indicate absence of the nanophotonic structure):

$$\frac{I}{I_{\text{ref}}}=\frac{P}{P_{\text{ref}}}\times \frac{QE}{{QE}_{\text{ref}}}\times \frac{C}{C_{\text{ref}}}.$$

As long as the emitter is not saturated, the pump enhancement *P*/*P*_ref_ simply maps the local pump-field enhancement at the structure. The quantum efficiency can be modified due to LDOS changes, while the collection efficiency *C* is influenced by the emission directivity induced by a structure.

**Excitation enhancement** When considering pump field enhancement, for optically driven fluorophores, a nanophotonic resonator can significantly enhance pump rates if the pump light is chosen to be resonant in frequency with a mode of the resonator. Particularly for plasmonic resonators, such as metal nanoantennas, this leads to strongly spatially varying pump field distributions (see Figure 4b for the nanocube-on-mirror example). For instance, reported pump field enhancements in bow tie gap antennas and nanocube-on-mirror constructs are of order 100–500× [21, 22, 106], confined to volumes no more than 10 nm across. These distributions arise as the coherent sum of the input beam, and the scattered pump light, which generally has both propagating components and sharply confined near-field components.

**Local Density of States** For understanding light–matter interaction in the weak-coupling regime, the so-called local density of optical states or LDOS is the central quantity [107]. Its roots go back to a seminal note by E. M. Purcell stating that the spontaneous emission rate of an emitter located in a cavity of mode volume *V* and quality factor *Q* is proportional to the so-called Purcell factor [108]. This has later turned out to be a specific example of the more general fact that spontaneous emission rates can be enhanced in proportion to the local availability of electromagnetic modes [103]. Qualitatively, the idea is that the emission rate of a fluorophore is set by Fermi’s Golden Rule to be proportional to the number of final states to decay into. This includes both final electronic states for the emitter as well as available states for the emitted photon. On this basis, Fermi’s Golden Rule can be rewritten to

(1) $$\gamma =\frac{\pi }{3\hbar }|\mu {|}^{2}\rho_{\mu }\left(\omega ,r\right),$$

where *ρ*_*μ*_(*ω*, **r**) is the local density of states (LDOS), which gives the number of states per unit volume and unit frequency at frequency *ω*, available to an emitter at position **r** with its dipole moment oriented along ***μ***. For dielectric systems, such as photonic crystals, the LDOS can literally be calculated by enumerating eigenmodes of Maxwell’s equations through *∑*_all modes *n*_| ***μ*** ⋅**E**_*n*_(*ω*, **r**)|^2^*δ*(*ω* − *ω*_*n*_) (mode eigenfrequencies *ω*_*n*_ and field profiles **E**_*n*_(*ω*, **r**)). For photonic systems with absorptive and dispersive constituents, e.g. for emitters near metal nanostructures, the LDOS can be calculated through the imaginary part of the Green function [107]. Importantly, the LDOS is a quantity that is strongly dependent on frequency and on spatial position. A main challenge in nanophotonics in the context of light–matter interaction enhancement is to identify the location of highest LDOS and to place the emitter there.

Super-resolution imaging of fluorescent decay rates can resolve LDOS at the nanometer scale. The idea is that the fluorescent decay rate of a fluorophore changes with LDOS as:

(2) $$\gamma =\gamma_{\text{nonrad}}+\gamma_{\text{rad}}{\tilde{\rho }}_{\mu }\left(\omega ,r\right),$$

where $\tilde{\rho }$ represent the LDOS relative to that in vacuum, and according to the rationale that only the intrinsically radiative processes are susceptible to the electromagnetic mode density. Measured changes in the total fluorescence decay rate thus translate into a measurement of the LDOS, on the proviso that the intrinsic radiative and nonradiative decay rates are calibrated for the emitter *a priori*. Finally, it is important to understand that the accelerated decay $\gamma_{\text{rad}}{\tilde{\rho }}_{\mu }\left(\omega ,r\right)$ itself contains both radiative and nonradiative contributions. For absorbing photonic structures, such as plasmonic antennas, both excitation of plasmon resonances that are Ohmically damped as well as non-resonant energy transfer to the metal contribute to fluorescence quenching. This can be captured by a separation of the LDOS into individual contributions, for instance as

$${\tilde{\rho }}_{\mu }\left(\omega ,r\right)={\tilde{\rho }}_{\mu ,\text{free space}}\left(\omega ,r\right)+{\tilde{\rho }}_{\mu ,\text{guided}}\left(\omega ,r\right)+{\tilde{\rho }}_{\mu ,\text{loss}}\left(\omega ,r\right).$$

for photonic systems with a guided mode (e.g. waveguides, surface plasmon polaritons, nanowire). Into what channels one has to separate the LDOS depends on the problem at hand. For instance, for plasmonic antennas there is no guided mode, and the interest lies in maximizing ${\tilde{\rho }}_{\mu ,\text{free space}}\left(\omega ,r\right)$. In this picture, the ultimate system’s quantum efficiency is modified by the nanophotonic structure, and becomes in itself a quantity of interest to resolve. Figure 4c and d illustrate the LDOS and its decomposition into radiative and nonradiative contributions for the case of a nanocube-on-mirror patch antenna. Over nanometer length scales, the LDOS can vary by three orders of magnitude.

**Directivity** Modifying the radiation patterns of light sources in nanostructures is in itself an important goal of nanophotonics. Control of emission directivity of single emitters such as quantum dots, organic molecules, or solid-state color centers is pursued for their applications as single photon sources with improved photon collection efficiencies [18, 20]. In a parallel development, plasmonic and dielectric arrays and aperiodic structures have been demonstrated to be highly effective for redirecting emission of ensembles of emitters, with possible uses for remote phosphors in solid-state lighting [9]. For fluorescence microscopy, directivity enhancements by nanoantennas can significantly boost count rates per molecules [109, 110]. Finally, directivity in emission is reciprocal to directivity in absorption at the same wavelength [18]. Controlling directionality of absorption and emission is a main goal for improving nanostructured photovoltaic devices [111]. There are few main mechanisms that can generate directional emission. One limiting case is when a light source is coupled to a nanophotonic resonance with a high Purcell factor. The high Purcell factor signifies that the light source will mainly emit via exciting the resonator mode, which in turn means that the far-field radiation is essentially distributed along angles of the radiation pattern of the resonator eigenmode. Thus, the radiation pattern of a nanosource that is efficiently coupled to, say, a plasmonic nanorod resonance, can be essentially dominated by the electric dipole emission pattern of the nanorod. More generally, in resonant nanophotonics an emitter is coupled to several resonances at the same time, such as the electric and magnetic multipolar resonances of Mie scatterers or plasmonic oligomers [112]. In this case, the radiation pattern is the coherent superposition of the direct dipole emission that reaches the far field, and that of the induced multipolar resonances. This interference mechanism underlies directional emission based on Kerker-effects in light emitting metasurfaces. Multimode interference does also occur for nanoparticle-on-mirror patch antennas, in which electric dipole, magnetic dipole, and quadrupole modes participate (for an example, see Figure 4e).

Finally, diffractive effects and phased array antenna physics can cause strongly directional emission. Diffractive directional emission generally operates by first funneling emission preferentially into a waveguide mode, and subsequently outcoupling the waveguided emission via diffraction [9]. In plasmonic and dielectric systems with surface lattice resonances, the emission behavior is determined by an interplay of grating diffraction on one hand, and multipolar interference on the other hand. Thereby, directivity can sensitively depend on source location, as source location determines the relative amplitude and phase with which the multipolar resonances in a structure are excited. Unraveling the physics of directivity control does therefore require the measurement of radiation patterns with Fourier microscopy of individual nanosources that are pinpointed in space by a super-resolution technique. It was recently shown that this idea can even be reversed: once a library of radiation patterns as a function of position is measured, one can reconstruct the location of source to within 10 nm precision simply by analyzing its radiation pattern (radiation-pattern-based localization microscopy) [113].

## 4 Super-resolution imaging for nanophotonics: state-of-the-art examples

As detailed in Section 3, one of the main challenges for probing light–matter interaction in nanostructured materials is the measurement of the excitation enhancement, LDOS modification, its radiative and non-radiative contributions, and the radiation pattern. Even irrespective of the aims of nanophotonics for controlling light–matter interaction, super-resolving electromagnetic field distributions in driven nanophotonic structure has been a main ambition for the community of near-field scanning probe microscopy since the mid-1990s [114], fueled by the emergence of systems with exotic electromagnetic modes, such as photonic crystal waveguides and cavities, surface plasmon polariton waveguides, or plasmonic antennas. Instead of using a comparatively bulky scanning probe (20–200 nm in size typically), a single emitter would provide the ultimate highest resolution in mapping such electromagnetic fields. This notion was developed in the near-field community, leading to fluorescent-probe near-field scanning microscopy [115]. Super-resolution localization microscopy provides a more facile realization of this idea in the sense that it removes the need to mechanically manipulate single-emitter near-field probes. Moreover, the presence of a scanning tip, which perturbs the environment, is not necessary, and single molecules can access regions of space which are not accessible with a scanning tip. The experimentally accessible quantities in SMLM or, more generally, super-resolved experiments, are fluorescence intensity and decay rate that are related to the physical quantities described in Section 3. In this section, we will review nanophotonics experiments based on super-resolution microscopy.

### 4.1 Intensity-based experiments

**Figure 5:**
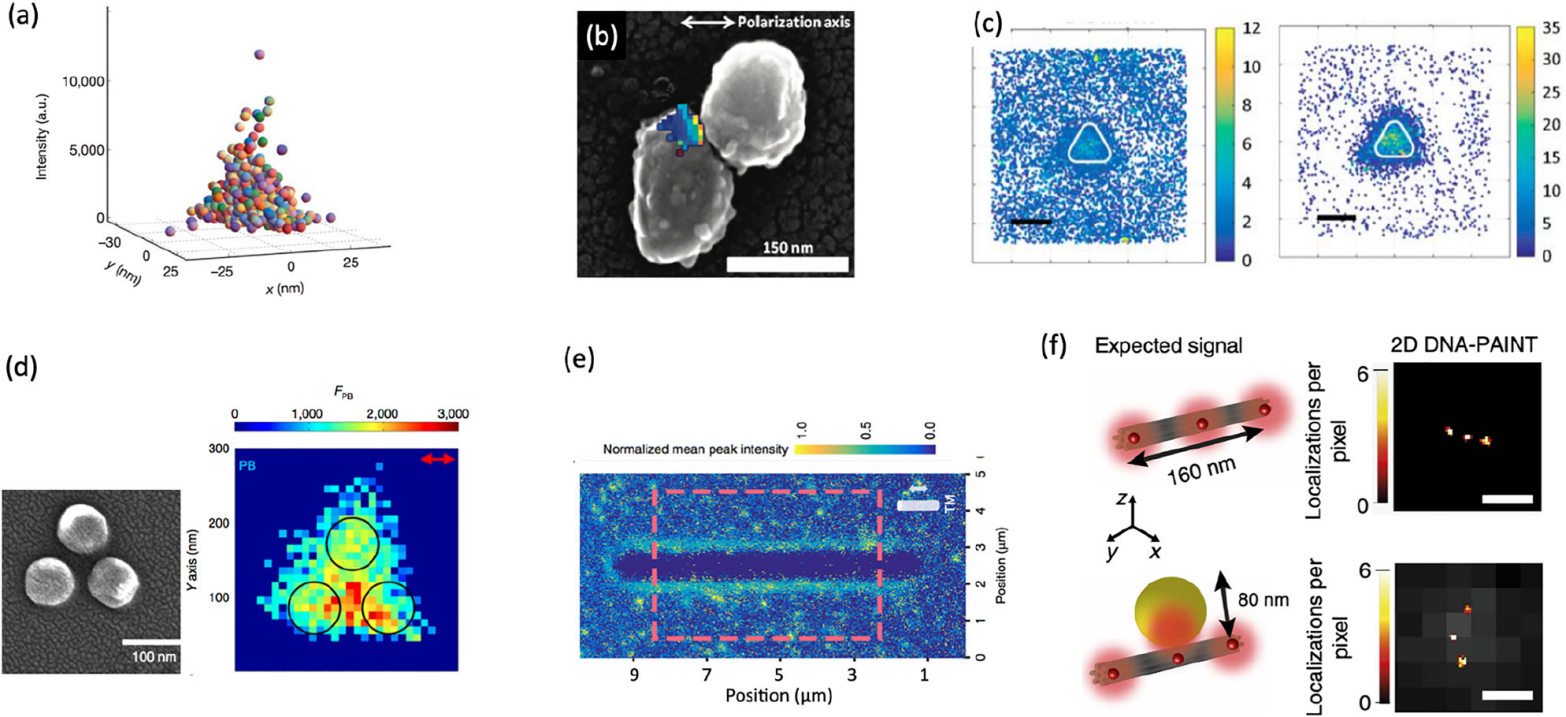
Intensity-based single-molecule experiments to study light-matter interaction at the nanoscale. (a) Super-resolved image of a hotspot on an aluminum film. Each dot indicates a single molecule detection. Adapted from [117]; (b) correlation between the SEM image and super-resolved fluorescence image of a colloidal silver structure for SERS. Adapted from [118]; (c) mapping of the fluorescence intensity of single molecules in the near-field of a nanotriangle for out-of-resonance (left) and in-resonance (right) conditions. Adapted from [122]; (d) SEM image of a three aluminum nanodiscs arranged on the apex of a triangle. Corresponding fluorescence intensity map obtained by single-molecule imaging of single molecules adsorbing at the surface of the structure. Adapted from [123]; (e) normalized intensity map for single molecules adsorbing to the surface of a 75 nm diameter silicon nanowire. Adapted from [124]; (f) DNA-PAINT image of three binding sites located at a distance of 80 nm one to the other in the presence (bottom) and in the absence (top) of a gold nanosphere. Adapted from [125].

The most straightforward quantity to be measured in super-resolution single-molecule microscopy is the fluorescence intensity. Seminal experiments in this field have been carried out by Stranahan et al. [116] and Cang et al. [117] on SERS surfaces. Such surfaces show huge fluorescent intensity enhancements within sub-diffraction limited areas and are therefore currently used to amplify the Raman signal of single molecules. In [116, 117], single-molecules adsorb on the surface and their fluorescence intensity is measured before bleaching, allowing to localize them. The localization accuracy can be as good as 1 nm. The density of molecules in solution is adjusted so that there is only one single molecule per diffraction limited spot, similarly to what is done in PAINT microscopy (see Section 2 for a description of PAINT). A direct measurement of single hotspots with a lateral extension as small as 15 nm has been realized with this technique, as shown in Figure 5a [117]. The correlated study between super-resolved intensity imaging and SEM images of SERS substrates, as shown in Figure 5b, shed new light on the understanding of hotspot formation in aggregates of colloidal plasmonic nanoparticles and initiated an interesting debate in the scientific community about single-molecule localization errors in the near-field of resonant structures [118–120], as we will see in Section 4.3. Later on, the understanding of hotspot formation in gold nanorods was also enriched by the combination of SEM images, single-molecule localization microscopy and defocused imaging [121].

A similar method has been applied later on to study nanostructures with hotspots located at pre-determined positions such as metallic nanodiscs and nanotriangles [122, 126]. As shown by Wertz et al. [122], the fluorescence intensity is modified in the presence of the nanotriangle. By tuning the emitters in and out of resonance with respect to the nanotriangle, as shown in Figure 5c, it is possible to show that both the fluorescence intensity enhancement and the emission pattern change. The use of deterministic structures has put in evidence the presence of a mislocalization effect. Due to the near-field coupling between the molecules and the metal nanoparticle, the fluorescence emission diagram of the molecule is modified with respect to the diagram in the absence of the structure. This change is reflected in a shift between the actual position and the apparent position detected in the far field. The two cases reported in Figure 5c, show that, regardless of the resonant character of the structure and therefore the fluorescence intensity enhancement, the apparent position of the molecules is on top of the nanotriangle, where the quenching is maximum, meaning that the entire structure is emitting. The mislocalization issue has been pointed out at the same time by Ropp et al. who explained it as the result of the interference between the radiation emitted by the molecule and its image induced by the presence of the structure [127]. Some recently published methods that allow to associate the apparent position to the real position will be discussed in Section 4.3.

When a fluorophore is coupled to a nanostructure, not only the emission of the fluorophore is modified, but also its absorption due to the enhancement of the excitation field. far-field collection of the fluorescence intensity therefore provides mixed information of the enhancement of the excitation field and the local density of states at the emission wavelength, as pointed out in Section 3. Using molecules with a large Stokes shift allows to spectrally decouple the emission of the molecule from the nanoantenna while leaving the absorption resonant. Based on that idea, the experiment realized by Mack et al. [123] provides a way of linking the fluorescent enhancement values with the electromagnetic field enhancement. The structure under study, shown in Figure 5d, is composed of three aluminum disks and shows a well-defined resonance. The fluorescence intensity image, obtained with PAINT microscopy, shows the presence of an intensity hotspot in the middle of the disks, in good agreement with numerical expectations.

Single-molecule microscopy has also been used for the study of the emission intensity enhancement and directivity modification in dielectric nanostructures, such as a 75 nm diameter silicon nanowire [124]. The fluorescence intensity map, measured via photo-activated fluorescent molecules in a liquid-phase medium adsorbing to the surface of interest, is reported in Figure 5e. The comparison of the data with analytical modeling allowed the authors to distinguish between the relative contributions of the different decay modes for different emitter dipole orientations [124]. In a similar way, the comparison between numerical simulations and experiments was used by [128] to disentangle the contributions of localized surface plasmon modes and of lattice surface modes in a hexagonal array of sub-wavelength aluminum nanostructures with a periodicity of 450 nm.

As already highlighted, SMLM suffers from mislocalization artifacts. Since the position where the molecule adsorbs to the surface is not *a priori* known, recovering its real position from the apparent position is challenging. A clever way to circumvent this problem is to use DNA-PAINT in which the molecules can only bind to specific binding sites located at a predetermined position. This is the method used by Raab et al. [125] who employed a rod-shaped DNA origami with three specific binding sites located at a distance of 80 nm one from the other. A gold nanoparticle (diameter 80 nm) sits in proximity to the central binding site. Figure 5f shows, in a striking way, a comparison between the localization of the molecules in the absence and in the presence of the gold nanosphere. While the three binding sites are perfectly aligned in the absence of the nanosphere, the central spot is misplaced in the presence of the sphere, due to the resonant interaction. The drawback of this technique however is that it cannot be used for mapping densely labeled samples.

With a completely different approach, using molecular motors or microfluidic chambers allows having deterministic information about the position of a single emitter in close proximity of a nanostructure [129, 130]. Such approaches, that benefit from an *a priori* knowledge of the emitter’s position, will be described in Section 4.4.

### 4.2 Lifetime-based experiments

As shown in the previous section, it is possible to monitor light–matter interaction by measuring the modification of the intensity emitted by a fluorophore when it is close to a nano-structured environment. However, intensity measurements are not fully reliable, because they depend on the excitation modification due to the nanostructure, to the presence of non-radiative modes, to blinking and photobleaching. Another quantity that can be used to measure light–matter interaction is the fluorescence decay rate, which is much more robust than fluorescence intensity because it is independent on the quantities listed above. Moreover, its direct relation with the LDOS, shown in Section 3.2, opens the possibility of measuring this quantity without the need of numerical simulations.

**Figure 6:**
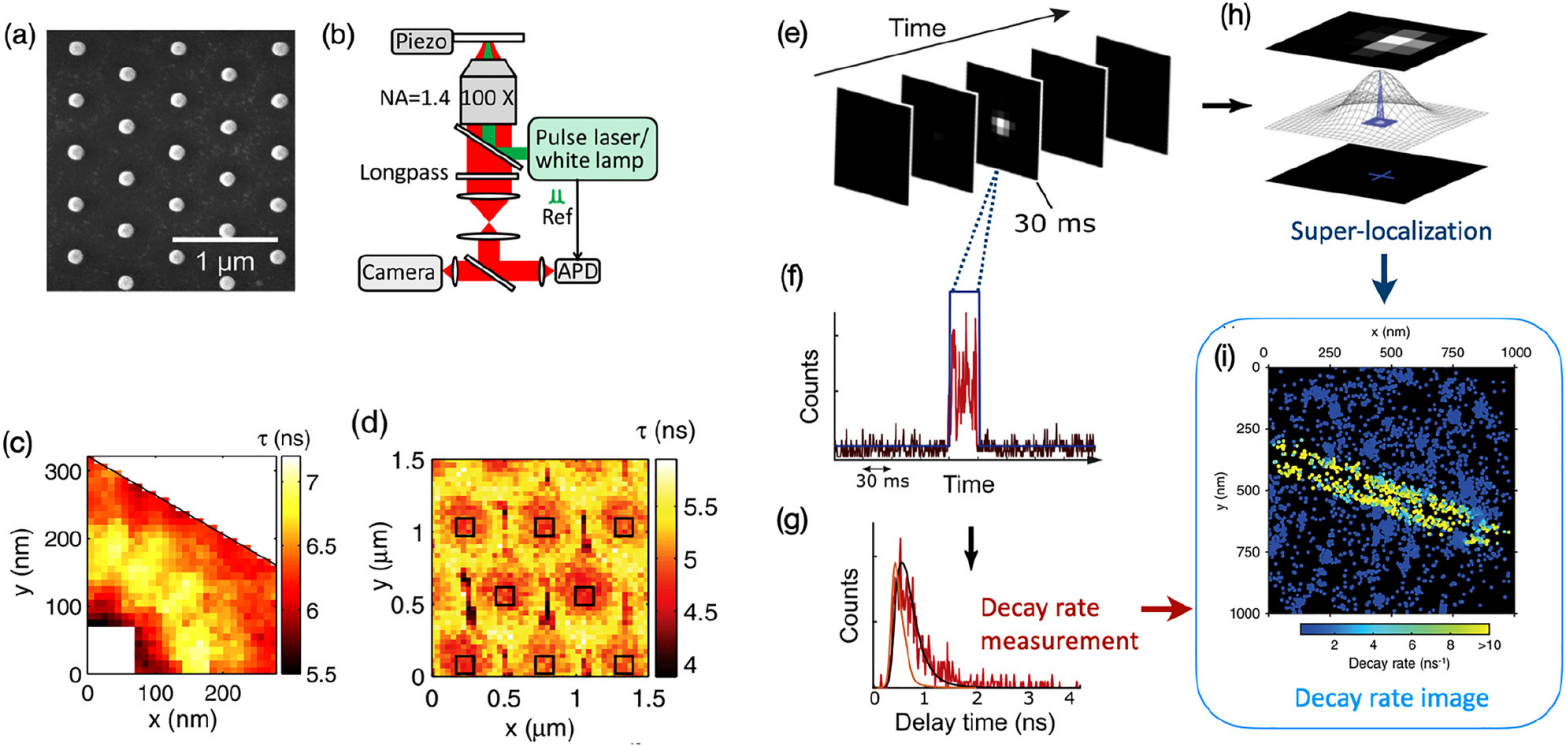
Lifetime-based single-molecule experiments to study light-matter interaction at the nanoscale. (a–d) Super-resolved imaging of the decay rate in the near-field of a lattice of aluminum nanoantennas, adapted from [131]. (a) SEM image of a hexagonal lattice of aluminum nanoantennas. (b) Sketch of the experimental setup. (c) Fluorescence lifetime map, obtained with 40 nm fluorescent spheres randomly spread on the sample, of the unitary cell around a nanoantenna. The white square delimits the boundary of the nanoantenna. (d) Fluorescence lifetime map obtained with a SNOM tip, adapted from [131]. (e–i) Illustration of smFLIM applied on a silver nanowire, adapted from [132]. A sequence of EM-CCD frames is acquired (e). The bright spot in the central frame is the PSF of a single molecule. The PSF is fitted with a Gaussian fit and its center is located with a precision of about 10 nm (h). Simultaneously, half of the photons emitted by the molecule are detected on a time-resolved SPAD and a TCSPC device allows to extract the decay rate of the fluorescent emission (f and g). By combining the information on the position and decay rate of each molecule one can reconstruct the decay rate image in the near-field of a silver nanowire (i).

Taking inspiration from super-resolution microscopy techniques developed for biophysics applications, Guo et al. developed a method which combines decay-rate measurements with a TCSPC system and emitter localization by fitting the PSF [131]. By comparing the results obtained with stochastic fluorescence microscopy, a state-of-the-art SNOM setup and FDTD simulations, Guo et al. performed a thorough study of hexagonal array of aluminum nanoantennas. Such structures have interesting application for light extraction from LEDs because they couple light to well-defined direction. The SEM image of the studied array is reported in Figure 6a. For the application of the stochastic microscopy method, some fluorescent spheres with a diameter of 40 nm are spread on the sample, in such a way that their separation is larger than the diffraction limit. This allows to fit the PSF of each bead and to localize it as it is done with single molecules in single-molecule localization microscopy. Since the studied structure is periodic, fluorescence collected from different regions of the array can be reported on the unitary cell and averaged to reduce the statistical error, smooth the differences between different nanoprobes and different nanostructures. The lifetime of each probe and its position is simultaneously measured by splitting the fluorescence photons into two paths, one being detected by an EM-CCD camera to measure the position, the other by a SPAD for time-resolved measurements, as shown in Figure 6b. The lifetime map obtained with this method is reported on Figure 6c. The observed lifetime modification is then related to the LDOS. The resolution obtained with this method is 40 nm, which is the size of the fluorescent bead. The map obtained with this novel method is compared with state-of-the-art SNOM measurements. In this case, the sample is covered with a fluorescent polymer and a metallic coated SNOM tip with an aperture of 100 nm is approached to the surface. The excitation laser is then injected in the SNOM tip and the fluorescence is collected by the microscope objective under the sample. In the case of the SNOM setup, the position of the tip is *a priori* known with nanometric precision and the topography of the sample can be acquired together with the fluorescence intensity and lifetime. The fluorescence lifetime map is reported in Figure 6d. In comparison with the lifetime map obtained with the first method, the SNOM lifetime map shows a reduced contrast, due to the worse resolution. For the same reason, some details are washed out due to the lack of resolution. Moreover, the measurements are affected by some topographic artifacts and are not exempt from LDOS variations induced by the presence of the metallic coated tip in close proximity of the sample. FDTD simulations confirm the validity of both methods.

A few years later, Bouchet et al. pushed at the single-molecule level the combination of stochastic microscopy and lifetime imaging by applying it to a sample densely labeled with photoactivatable single molecules [132]. The advantage of this method with respect to the previously described one, beyond the fact that it is at the single-molecule level, is that it can be applied to the study of any structure, no matter if it is deterministic, periodic or random, metallic or dielectric.

The method, called smFLIM for single molecule Fluorescence Lifetime Imaging Microscopy, relies on the direct and simultaneous measurement of the fluorescence intensity and decay rate of stochastically fluorescent single molecules. The experimental setup is similar to the one used by [131] sketched in Figure 6b. The sample is excited in wide-field and single-molecule fluorescence is simultaneously detected on both an EM-CCD camera and a single-channel SPAD. The SPAD field of view is set to 1 μm^2^. By setting the excitation and photo-activation laser power so that no more than one molecule is active at a given time on the area conjugated to the SPAD, the decay rate can be properly estimated for each individual molecule and can be associated to its position. The same experimental parameters allow to set the bleaching time of each molecule to about 30 ms. The method is summarized in Figure 6e–h and the decay rate image obtained in the near-field of a silver nanowire (diameter 115 nm) on a glass coverslip is reported in Figure 6i. The emission of a single molecule is detected on an image or a sequence of images acquired with the EM-CCD (see Figure 6e) and a two-dimensional Gaussian fit is applied on the PSF in order to localize the emitter (see Figure 6h). The fluorescence of each molecule is also detected on the SPAD as a time-burst (see Figure 6f). The TCSPC system to which the SPAD is related, allows to measure the delay between the arrival time of the fluorescence photons on the detector and the time at which the molecule has been excited by a pulsed excitation. The decay histogram is reported in Figure 6g. A mono-exponential fit of the decay histogram allows to recover the decay rate of the emitting molecule. By associating the information on the position obtained with the EM-CCD and the decay rate one can obtain a decay-rate map as the one reported in Figure 6i, in the near-field of a silver nanowire on a glass coverslip. Each point on the map represents a single-molecule detection and its color the decay rate. The size of the points is the average localization precision, which is about 15 nm. As expected, the decay rate is enhanced close to the nanowire due to the presence of non-radiative decay channels. The decay rate on the nanowire is enhanced by a factor 15 with respect to glass. By taking a closer look to the decay rate map, one can notice that there are almost no events detected on the top of the nanowire, due to the inhomogeneity of the excitation field. Since the polarization of the excitation field is perpendicular to the nanowire, the intensity of the excitation field is enhanced on the sides of the nanowire and almost zero on top. Therefore, the molecules located in the lower excitation intensity regions have a very small probability to be detected, supporting the observed variations of the density of detected molecules. FDTD simulations support the observations. Further developments of this technique allowed to increase the field-of-view from 1 μm^2^ to 10 μm^2^, thanks to the use of a 8 × 1 SPAD array [133]. More details about this experiment will be given in Section 4.4.

### 4.3 Challenges for single emitter nanophotonics experiments

Super-resolved microscopies of nanophotonic structures using localization of single emitters face several main challenges. Beyond all the experimental challenges associated with single emitter photophysics near nanophotonic structures, there are important artifacts in single molecule localization that are specific to localization-based super-resolution applied to resonant environments.

As regards the general challenges for single emitter experiments in nanophotonics, these have largely been reviewed in many works, such as the reviews in refs. [18, 20, 91] and largely originate from two distinct reasons. First, the primary photophysical observables, such as brightness, are intrinsically due to the product of several different mechanisms, including pump rates, the collection efficiency of emitted photons, and the quantum efficiency of emission. Decomposing this product in its individual contributing terms is a major challenge for which a sequence of experiments is needed. For instance, varying the pump wavelength and geometry serves to decouple pump and emission effects, while comparing cw and pulsed excitation, and bringing emitters into saturation helps to obtain quantitative efficiency metrics. These ideas have also found use in the super-resolution microscopy field applied to plasmonics. Notably, Wertz et al. [122] pioneered the idea of using pump wavelength diversity to unravel pump enhancements and LDOS effects when interrogating resonant plasmonic nanotriangles. The second challenge resides in the calibration of the intrinsic photophysical properties of each single emitter. Indeed, this is the condition to fulfill to ensure that single fluorophore experiments accurately report on nanophotonic properties. For instance, a main complication for determining LDOS values is that the quantum efficiency of the reporting emitter must be accurately known. Calibrating such quantum efficiencies is challenging, especially for single emitters at a time [92, 96, 98], [99], [100], [101], [102]. Moreover, one often relies on the assumption that the quantum efficiency can be calibrated on emitters of the same type, without the nanophotonic structure, as opposed to really calibrating it for the very same emitter. Similar calibration considerations apply to dipole moment (absorption and emission transition dipole moments, static and dynamic orientation properties) and the spectral properties of the emitter (homogeneous versus inhomogeneous linewidths).

In the specific case of SMLM, a major challenge that experimentalists have to face is localization artifacts. While the first reports on super-resolution mapping of plasmonic hotspots, particularly SERS hotspots, pointed at localization errors as small as 5 nm [118, 120], systematic and significant sources of mislocalization were soon discovered and systematically analyzed [122, 124, 134], [135], [136]. These studies encompass a diversity of nanophotonic systems, from colloidal plasmonic particles and oligomers, to dielectric nanowires, and a diversity of single molecule localization strategies, and yet paint a coherent picture of mechanisms behind major artifacts [137]. These mechanisms are fundamental to the main photonic properties that super-resolution microscopy seeks to spatially map.

A main purpose of nanophotonic resonant structures is to locally provide high local density of optical states (LDOS), which is achieved by ensuring that light–matter coupling is dominated by just one or a few resonant modes. This directly implies that in this limit the emission can no longer be pinpointed to the emitter, and instead inherits the apparent spatial profile (in real space imaging) and radiation pattern (in Fourier imaging) of the resonator. If the resonance is, for instance, a dipolar plasmonic scatterer, this limit implies that any molecule driving the antenna appears to be located *at* the antenna center as can be seen in Figure 5c. However, PSF fitting to resolve molecule locations showed an apparent surplus of molecules exactly *on* the nanotriangle, and a clear deficit in a 50 nm shell around the nanoparticle. Consequently, localization artifacts are of the order of the particle size, and far exceed the artifacts expected merely from the photon budget. In this picture, it is evident that the nature of the resonator is of main importance.

**Figure 7:**
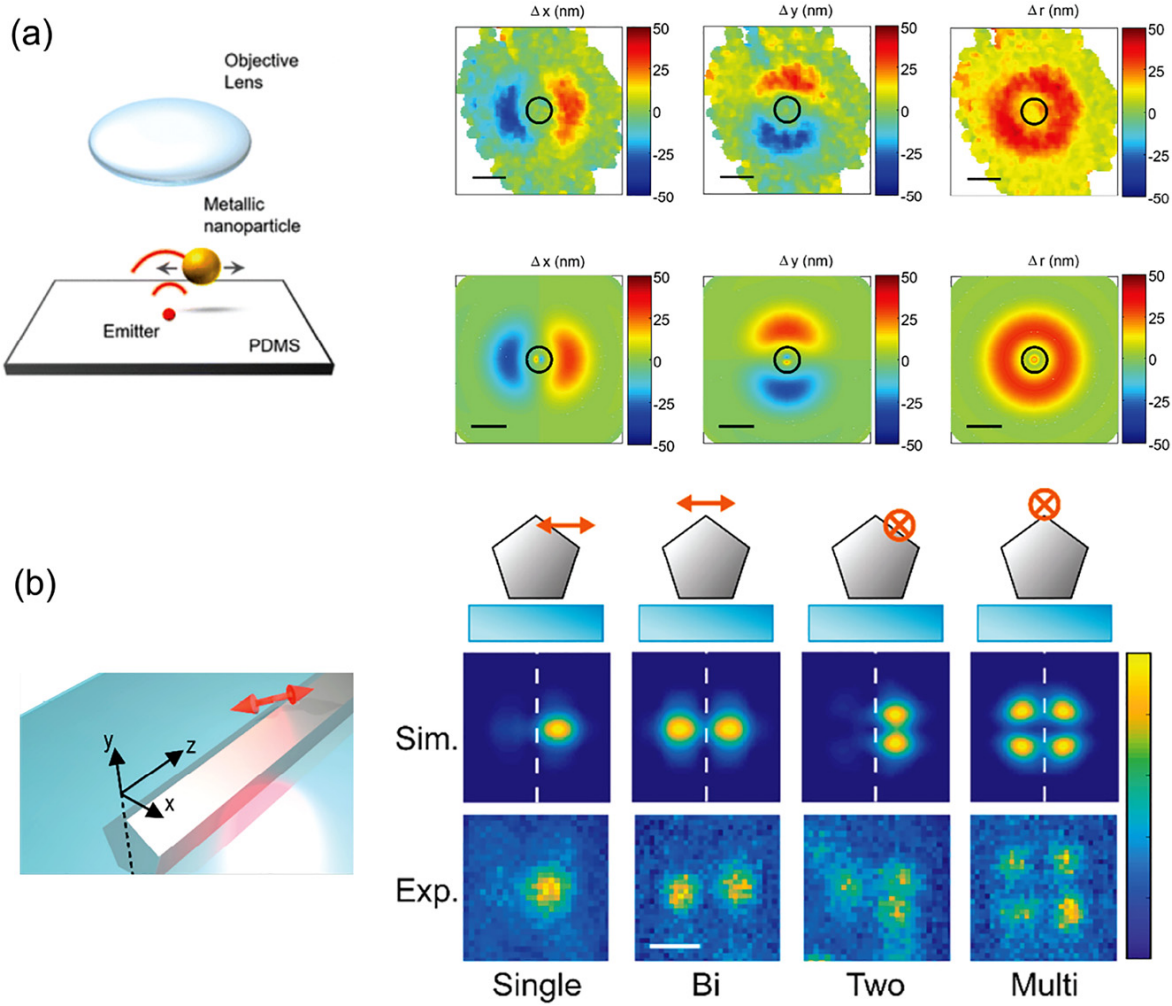
Evidence and analysis of systematic errors in superresolution localization microscopy due to strongly scattering nanophotonic environments. (a) Lim et al. [136] used an immobilized emitter in microscope focus while tracking a plasmonic nanoparticle moving around it in order to have a ground truth against which to benchmark PSF-based emitter localizations. The panels show as color scale the difference between retrieved and ground truth coordinate (along horizontal *x*, *y*, and total distance *r*) in measurement and according to simulation. Scale bars are 200 nm. Adapted from ref. [136]. (b) Baiyasi et al. [139] provide clear observation of the dramatic change of PSFs for emitters near a nanostructure. Instead of simple Gaussian-like PSFs this includes multi-lobed PSFs. Calculations show similar PSFs, which vary rapidly with molecule position and dipole moment orientation as indicated. Adapted from ref. [139]

In reality, the limit in which the nanophotonic system completely dominates the LDOS is almost never attained, and certainly not in the tails of the resonant near field. Instead, the nanophotonic system captures and subsequently re-radiates into the far field just a fraction of an emitter’s emission, while the remaining fraction is radiated directly by the emitter into the far field. The direct radiation from the emitter, and the radiation that reaches the detector only via the nanophotonic system, are coherent to each other, and hence interfere on the detector. Indeed, this spatial coherence is the main mechanism behind the fact that spontaneous emission directivity can be controlled by phased array nanoantennas and diffractive structures. The implication is that the radiation pattern (in Fourier microscopy) will show an interferometric signature of the superposition of direct dipole emission and the emission of the excited resonances in the nanophotonic system, with relative phases that depend on the position of the emitter relative to the structure. Turning to real space images instead of radiation patterns, this is equivalent to saying that the apparent PSF may significantly change as function of geometry, and it certainly must no longer be centered on *either* the molecule *or* the nanophotonic resonator. Figure 7 highlights both effects. A particularly systematic study of the mislocalization of emitters on the basis of the PSF was provided by Lim et al. [136], from which Figure 7a reports the salient result. The authors used an immobilized quantum dot as source, and a microfluidic system with a plasmonic nanoparticle moving through the solution as nanophotonic resonator. The advantage of this approach is that the emitter location can be fixed first in absence of the nanoparticle, while the nanoparticle can be tracked by its scattering with high accuracy independent of the fluorescence emission photophysics. This provides a ground truth with which to compare apparent emitter-particle distances as determined from fluorescence PSF analysis. Figure 7a shows emitter mislocalization (both coordinates *x*, *y* and overall position) as a function of this ground truth, highlighting systematic deviations up to 50 nm in a ring of around 100 nm radius around the nanoparticle. Numerical simulations reproduced these measured systematic artifacts. The significant shape changes of the PSF near nanostructures were thoroughly studied in the specific case of a silver nanowire in [138] and subsequently used by Baiyasi et al. [139] to associate the measured PSF to a specific orientation of the dipole moment. Figure 7b illustrates this effect. The experiment at hand concerns emitter localization near Ag nanowires of pentagonal cross section. The authors consider both emitter position around the wire circumference, and dipole orientation, and find in calculations that a multitude of PSF patterns can occur that are single-lobed, double-lobed, or even have four lobes. These patterns have indeed been identified in experimental data.

Aside from the artifacts that directly arise from nanophotonic LDOS and directivity control mechanisms, localization can also suffer from secondary effects that are tied to the luminescence properties of the emitter at hand, and of the nanophotonic resonator. First, many nanophotonic resonators themselves create background luminescence. This (generally spatially structured) background can impair fit accuracy. Second, a more treacherous problem comes from the fact that emitters of different quantum efficiency exhibit different brightness values in nanophotonic systems. High quantum efficiency emitters generally can only go down in quantum efficiency when approaching a nanophotonic resonator, and any brightness change is either due to quenching, or to local pump field enhancement. This should be contrasted to low quantum efficiency emitters, which in a high LDOS environment can show boosted quantum efficiencies. At the least, this can imply a bias in the probability that emitters are identified, as function of their proximity to a nanophotonic structure. For localized molecules, this can furthermore introduce biases towards sampling only certain transition dipole moment orientations. The fact that the near-field resonances that enter the LDOS do generally have strongly preferential electric field orientations directly translates into a strong dipole-moment orientation dependence of the LDOS. As a consequence, some orientations can give rise to much brighter emission intensities than others. To understand this bias, it must be clarified whether the experiment at hand samples with random dipole moment orientations, or whether is uses dipole emitters with preferential orientations.

### 4.4 Overcoming localization-artifacts

**Figure 8:**
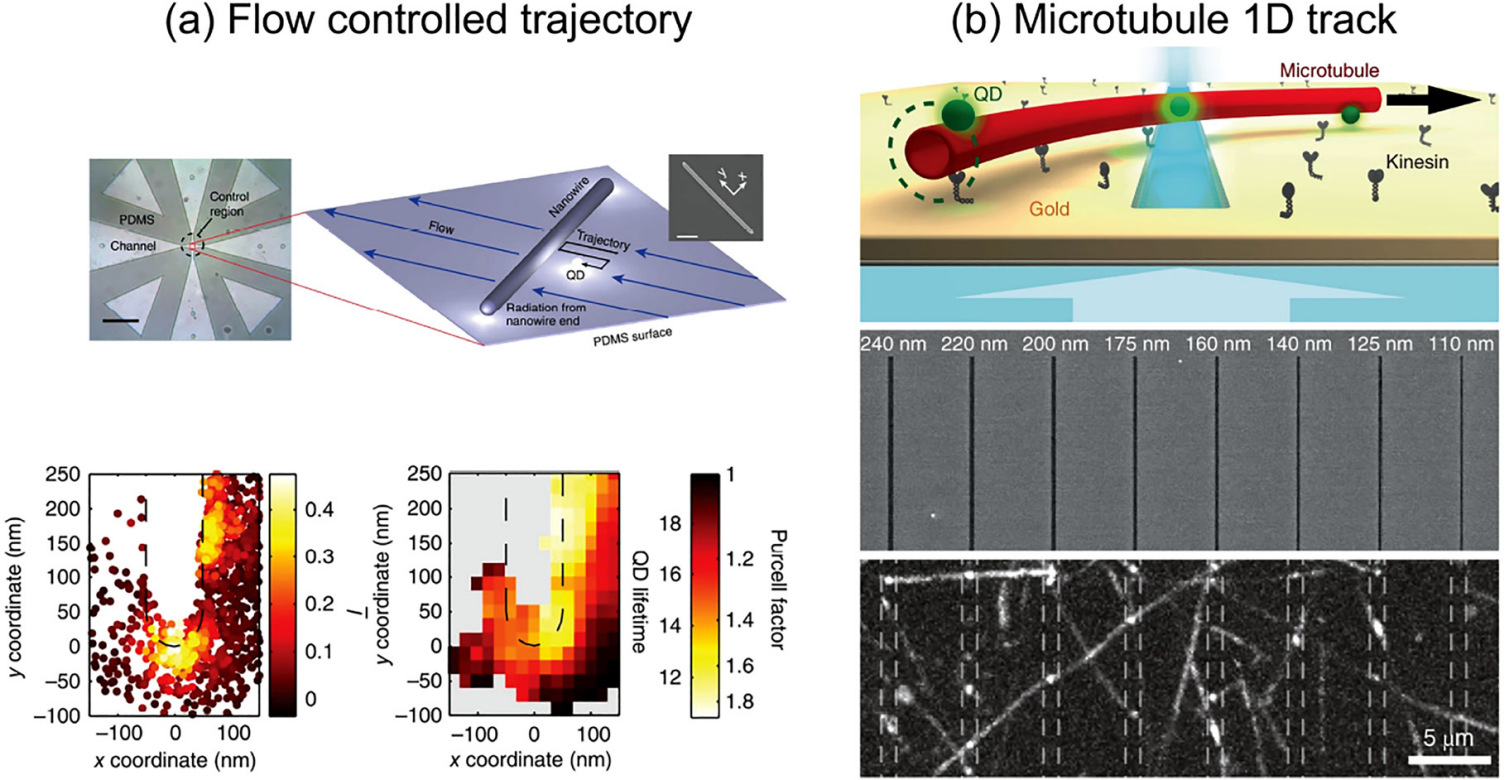
Strategies to improve super-resolution mapping of nanophotonic structures. They include microfluidic flow control (a) to controllably move quantum dots over a nanophotonic system, and (b) constraining sources to move over 1D tracks by attaching quantum dots to motor proteins that walk over microtubules. The method in (a) approaches the notion of a controllable scanning source, as envisioned originally in the NSOM community. The results [130] show mean fluorophore brightness $\bar{I}$ and the quantum dot decay rate as function of position next to a plasmonic nanowire (photons are collected from the nanowire ends). The approach of Groß et al. [129] leverages the fact that quantum dots constrained to 1D trajectories, defined by randomly deposited microtubules, can be accurately tracked. The sample in this work consisted of 1D slits in a metal film, illuminated from below and imaged from above. This method is mainly suited to measure the nanophotonic properties of translation invariant systems across 1D cross sections. Panels adapted from [129, 130].

We identify three classes of strategies to deal with and to overcome mislocalization artifacts. First, from a purely experimental perspective, several groups have deployed methods that avoid the need to localize completely randomly located fluorophores, using some mechanism to turn the challenge of localization into the more constrained and hence robust challenge of particle tracking. Figure 8 illustrates two successful strategies. Ropp et al. [130] developed a microfluidic platform in which the nanophotonic structure of interest is located on a PDMS substrate, surrounded by four fluidic inlets. Microfluidic flow control is then used to position and track quantum dots with about 35 nm positioning accuracy. It has been demonstrated that lifetime measurements can be used to probe the LDOS, as illustrated for silver nanowires. A different approach was developed by Groß et al. [129]. Their work used quantum dots attached to motor proteins walking over microtubule tracks deposited randomly over a plasmonic system. The crucial property of this approach is that the microtubules, due to their persistence length, strongly constrain the quantum dot motion along a well-defined 1D curve. By taking time-lapse recordings, one can track this motion, and hence improve localization accuracy. The disadvantage is that densely sampling a single nanostructure is difficult with this approach. Indeed, the idea was demonstrated on a translation-invariant slit system, where this approach yields 1D cross sections of the LDOS. A similar strategy has been adopted to study the intensity and lifetime modification of a single emitter walking into a plasmonic hotspot in the gap of a gold nanoantenna [140].

The second main strategy evident from literature is that a necessary condition for understanding and correcting localization artifacts resides on the detailed theoretical/numerical study of the nanostructure at hand. While it would be desirable for super-resolution microscopy to work independently on the details of the studied sample, it turns in fact out that it is mandatory to perform detailed simulations of the light–matter interaction to identify and to quantify the mechanisms that cause mislocalization artifacts. This includes full wave-optical simulations of the excitation electric field distribution, of the LDOS, of the radiation pattern and its relative orientation with respect to the objective’s cone of light detection, and of the final image formation on the microscope’s detector. Moreover, this full cycle needs to be done with including the vector properties of light (polarization of LDOS, emitter, and far field), emitter quantum efficiency, and emitter spectral properties. Excellent examples of this approach to elucidate mislocalization artifacts as well as the deconvolution of nanophotonic properties are provided in refs. [122, 124, 136, 137, 139]. An important caveat is that these calculations are not only time-consuming (large geometries, many source positions), but also that standard routines built into commercial electromagnetic solvers for calculating radiation patterns (i.e., near to far-field transforms) do generally not give correct results for stratified substrate systems. One option is to use simplified models, such as coupled dipole models to model emitters near plasmonic substrates. Another recommended approach is to use recently published routines for accurate near-to-far-field transformation in stratified systems, in combination with the quasi-normal mode formalism [88, 141].

Finally, the third main approach to overcome mislocalization artifacts lies in basing localization not just on fitting intensity images, but in using multiple observables as degrees of freedom on which to base localization. This essentially turns the very effects that invalidate standard localization procedures, i.e. effects such as LDOS changes, PSF changes, or radiation pattern changes, from a weakness into a strength. Blanquer et al. [133] showed that it is possible to correlate lifetime information (smFLIM measurements) and real space imaging for “relocating” molecule localizations to their correct position. In essence, the correlation of lifetime changes with the width of the observed PSF allows to pinpoint more accurately the dipole position and orientation of fluorophores near a nanophotonic structure of interest. This correlated approach thereby allows in turn a more precise reconstruction of LDOS maps. In a similar vein, one can consider to use radiation patterns as a proxy to guide localization. The strong dependence of radiation patterns on position is discussed in refs. [134, 142]. An implementation of the idea that this could be used for position reconstruction was recently reported by Buijs et al. [113]. In that work, the electron beam of a scanning electron microscope was used to generate luminescence that originates from a well localized spot (5 nm accuracy, from SEM image). Radiation pattern changes could be used to recover the position of the source to within 20 nm. That localization was “library-based” as opposed to physics-based: instead of using an *a priori* model, the method pinpointed the source locations by matching observed radiation patterns to a previously measured library of radiation patterns. These examples show that localization that is based on correlation of intensity, radiation patterns, decay rates and/or PSFs, interpreted either through physics models or, alternatively, supervised and unsupervised learning approaches, may reach single digit nanometer localization accuracy.

## 5 What nanophotonics can do for biophysics below the diffraction limit: state-of-the-art examples

Exploiting super-resolved techniques initially developed for biophysics has been a real benefit for nanophotonics. Reciprocally, nanophotonics effects have found application for studying biophysical problems. In this section, we will focus on two selected topics in this field. The first one concerns the nanometric axial localization of fluorescent emitters (i.e. determining the axial position of an emitter along the optical axis with nanometric spatial resolution), and the second topic concerns fluorescence-lifetime single-molecule localization microscopy (FL-SMLM or smFLIM) applied to biophysical systems.

### 5.1 Using near-field coupling for axial localization

**Figure 9:**
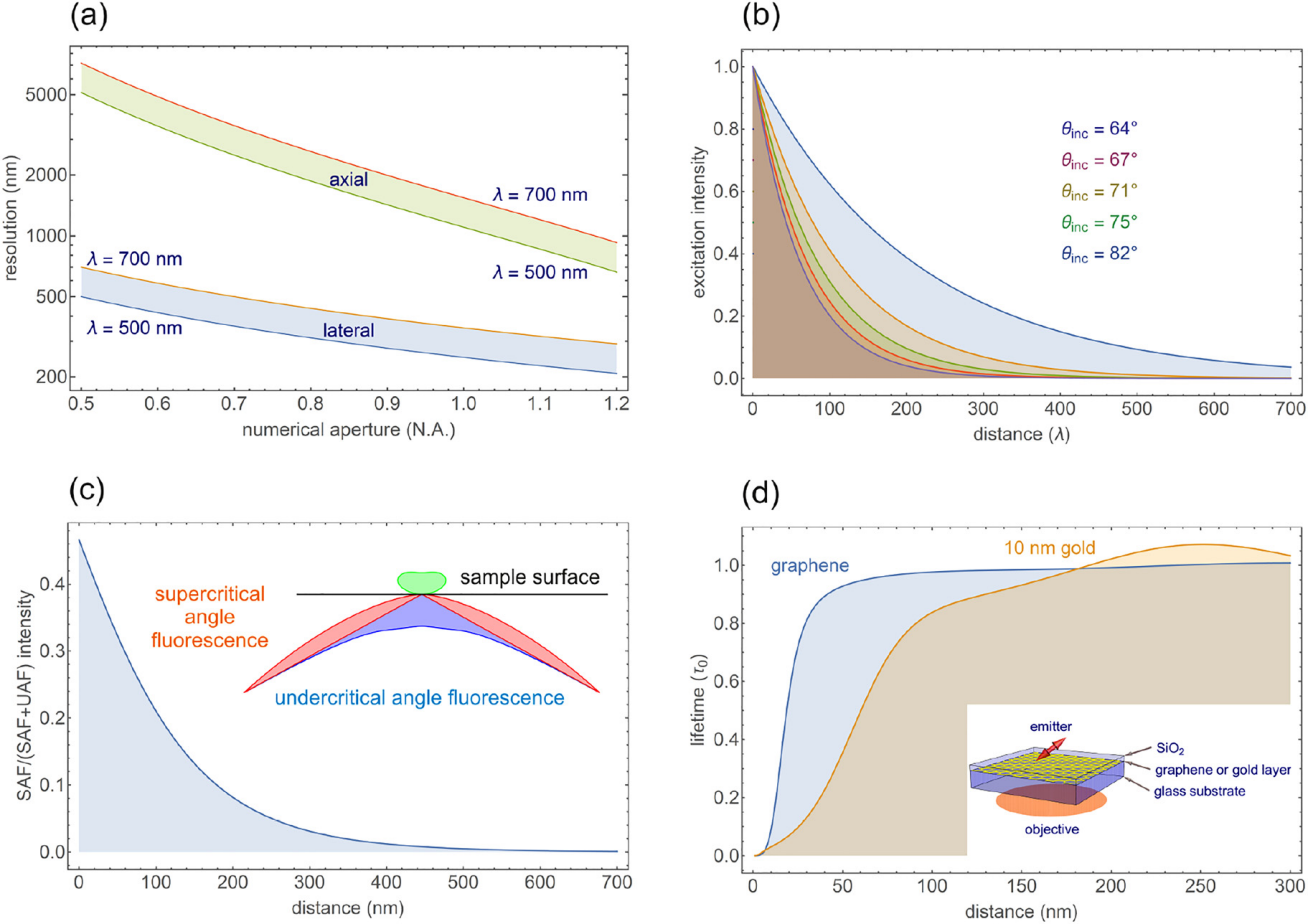
Axial resolution for 3D super-resolution microscopy. (a) Comparison of lateral (lower blue band) and axial (up-per green band) resolution in conventional diffraction-limited optical microscopy with a 1.2 NA water immersion objective. These resolutions are wavelength-dependent and the shown bands span a wavelength range between 500 and 700 nm. (b) Variable angle Total Internal Reflection Microscopy (vaTIRFM). (c) Super-critical Angle Fluorescence (SAF) imaging. (d) Metal-Induced Energy Transfer (MIET) and Graphene-Induced Energy Transfer (GIET) imaging.

As explained in Section 2, SMLM can achieve a lateral resolution that comes close to molecular dimensions. For example, in a seminal paper by the Sauer group [143] it was shown that dSTORM can resolve the molecular architecture of a nuclear pore complex. However, the big challenge is imaging the third dimension of real samples and correspondingly large efforts have been applied to achieve true three-dimensional super-resolution microscopy. The original STED microscopy focused only on lateral resolution, while more recent variants increased also the axial resolution by using modified phase plates [144]. Modern state-of-the-art STED microscopes deliver full 3D super-resolution by uniting two different setups (one for lateral, one for axial STED) into one single system [145]. For SMLM, several methods for 3D single-molecule localization have been developed, such as astigmatic imaging [146, 147], biplane imaging [148], or Point Spread Function (PSF) engineering [149, 150]. The combination of intensity and relative phase information can also be used to retrieve the axial localization thanks to a carefully engineered mask phase on the emission path of the optical set-up [151]. This lead to the recently introduced Self-Interference (SELFI) microscopy [152] that is capable of 3D imaging tens of microns deep inside a sample. However, all these approaches share one common characteristic: the achievable axial resolution is three to five times worse than the lateral resolution, similar to the situation in classical diffraction-limited optical microscopy, as shown in Figure 9a. Exceptions to this rule are interferometry-based techniques, where fluorescence is either excited and/or detected from two sides of a sample with two objectives, to either generate an axial excitation intensity interference pattern, or to interfere the collected fluorescence on a detector (such as for iPALM [153] and isoSTED [154]). Another recently described approach named ModLoc [155] relies on exciting the sample with a modulated interference pattern and measuring the relative phase between each fluorophore response and the excitation. This technique is robust to optical aberrations several tens of microns deep inside the sample and has shown axial localization accuracy of ∼7 nm without compromising in-plane localization. Another fascinating alternative for extreme 3D-resolution is the recently developed 3D-MINFLUX [156]. However, these techniques are exceptionally complex in their technical realization, which has prevented their wider distribution and application so far.

When interested predominantly in achieving high axial resolution close to a surface, near-field-based methods represent an attractive and usually simple to implement alternative for 3D-super-resolution microscopy with exceptional axial resolution. One of them is variable-angle total internal reflection fluorescence microscopy (va-TIRFM), which records several images at variable angle of excitation light incidence, which yields an evanescent excitation field (near-field) with different penetration depth into a sample [157–159], as shown in Figure 9b. By applying a suitable data analysis, it is then possible to calculate absolute distance values of fluorescent emitters from the different recorded images, but in practice, photobleaching does severely restrict the applicability of va-TIRFM to SMLM because several wide-field images have to be recorded for generating one 3D image.

Super-critical angle fluorescence (SAF) imaging [160–163] use the strongly distance-dependent near-field coupling of the electromagnetic field of an emitting fluorophore into a glass substrate (cover slip) for deducing the distance of an emitter form the surface, as shown in Figure 9c. However, both va-TIRFM as well as SAF-based methods are intensity-based methods and may suffer in performance when the refractive index of the sample is not well known or is variable, or under circumstances that skew the absolute brightness measurements required for their functioning.

An alternative near-field-based approach is Metal-Induced Energy Transfer (MIET) imaging [164], which is based on the strongly distance-dependent quenching of a fluorescent emitter in the vicinity of a metal, see Figure 9d, a phenomenon well-known by the nanophotonics community. This quenching is caused by the resonant transfer of the excited state energy of the emitter to surface plasmons (collective oscillations of free electrons) in the metal. This energy transfer leads to a distance-dependent modulation of fluorescence lifetime (and intensity) as shown in Figure 9d. Due to the broad absorption spectra of metals, the energy transfer from a fluorescent molecule to a metal film takes place with high efficiency across the full visible spectrum. Thus, any dye in the visible spectral range shows this effect, and its measured fluorescence lifetime can be converted into a distance of the emitter from the metal surface. The semi-classical quantum-electrodynamic theory for this process was worked out by Lukosz and co-workers [165, 166] and by Chance, Prock and Silbey [167] in the last century, and was brilliantly confirmed by experiments of Kuhn and Drexhage [168, 169]. Using this theoretical framework, it is straightforward to calculate MIET curves for an arbitrary configuration of planar layers (such as a glass substrate covered by a metal layer and an additional dielectric spacer, the typical configuration used in most MIET applications), and for fluorescent emitters of any emission wavelength (or emitters with broad emission spectra) and dipole orientation.

The first application of MIET imaging was the mapping of the height profile of the basal membranes of different cells [164]. In this study it was shown that MIet allows to profile cellular membranes with an axial resolution of 2–3 nm in a calibration-free and absolute manner. Since this first publication, MIET imaging was used in numerous biological studies. In ref. [170], MIET was employed to study the 3D architecture of focal adhesion complexes [170]. There, the authors performed MIET in two different spectral channels (dual-color MIET) which allowed them to co-localize in three dimensions different structures (actin, vinculin) in a focal adhesion. Another study followed the reorganization of the actin cytoskeleton during the transformation of epithelial to mesenchymal cells [171] over a time range of several hours. In ref. [172], MIET imaging was used to measure the inter-bilayer distance of a nuclear envelope, and ref. [173] used a phasor-based approach for MIET imaging to study the spatial organization of major nuclear lamina proteins. The authors of ref. [174] used the advantage of MIET that it does not require high-NA objectives for presenting a large field-of-view implementation of the method.

In 2014, it was demonstrated that MIET works even on the single molecule level [175], despite the unavoidable fluorescence intensity quenching by the metal layer. This is due to the fact that, although the fluorescence brightness of a dye is increasingly reduced the closer the dye comes to the metal surface, its photo-stability increases proportionally, so that the average number of detectable photons from one molecule until photo-bleaching is nearly independent of the dye-metal distance. This opened the way for using MIET as a tool for single-molecule localization along the optical axis. In a recent paper, proof-of-principle experiments were presented that demonstrated single-molecule co-localization along the optical axial of up to three emitters placed at well-defined positions on 3D-DNA-origami nanostructures [176].

Even for the most photostable dyes that can be used in SMLM of biological samples, the average number of detectable photons until photobleaching does typically not exceed a few thousand photons, which allows for an axial localization accuracy in MIET of a few nanometers, or a co-localization accuracy of ca. 10 nm. This is still bigger than what is required for resolving intramolecular details of medium-sized macromolecules (∼2–5 nm) or for distinguishing between the leaflets of a lipid bilayer. Here, materials with a much stronger quenching properties come to rescue. Already long before the introduction of MIET imaging, Hof and colleagues used the distance dependent fluorescence quenching by thin layers of indium tin oxide (ITO) for tuning the lifetime of fluorescent molecules, and to use this tuning in fluorescence lifetime correlation spectroscopy (FLCS) [177] for studying the diffusion and flip-flop dynamics of lipids in supported lipid bilayers [178, 179]. The advantage of ITO is that the axial range over which efficient fluorescence quenching occurs is much shorter than for MIET, due to the small imaginary part of its refractive index. In 2016, Moerland and Hoogenboom demonstrated that this short-range ITO-induced lifetime tuning can be used to axially localize single molecules with nanometer accuracy [180]. The disadvantage of ITO is that its exact dielectric properties (complex-valued refractive index) depend on the peculiarities of preparing the ITO layer on the glass substrate, and these properties can even change over time due to the diffusion of atmospheric oxygen into the material. As an alternative to ITO, other materials have been studied, and the most promising so far occurred to be single sheets of graphene. Graphene shows a similar short-range quenching as ITO [181, 182], see Figure 9d, but has highly reproducible and well-known dielectric properties. The authors of ref. [183] successfully used graphene-induced energy transfer (GIET) to measure the thickness of single supported lipid bilayers with sub-nanometer accuracy. In that paper, it was also demonstrated the GIET works perfectly well also for axial localization of single molecules with nanometer accuracy. This high axial resolution was used in ref. [184] for unraveling the complex association/dissociation dynamics of a membrane-associated protein complex. A global view on the huge possibilities and potential of GIET is presented in ref. [185], while a step-by-step protocol of how to set up and perform GIET measurements is published in ref. [186].

### 5.2 Fluorescence lifetime single-molecule localization microscopy for bio-imaging

By adding the lifetime dimension to the intensity information, FLIM has found many applications in bio-imaging: for example, for multiplexed imaging of fluorophores that are spectrally similar but differ in their lifetimes, for FRET imaging using the donor lifetime, for lifetime-based sensing in biological or environmental analytics, or for MIET/GIET imaging as explained in the previous section. The combination of FLIM with super-resolution microscopy, and in particular with SMLM, adds to the extremely high spatial resolution of SMLM the additional important information of fluorescence lifetime. As detailed in Section 4.2, the core idea of fluorescence-lifetime SMLM (FL-SMLM or smFLIM) is to measure the lifetime of each single-molecule localization event and to reconstruct a super-resolved image with this additional information. We identify two main experimental approaches for super-resolved imaging of the lifetime of single molecules. Such techniques suitable for SMLM are currently not widely available. In smFLIM techniques described in Section 4.2, photons emitted by a single molecule are simultaneously detected on an EM-CCD (for localizing emitter’s position) and a SPAD or a SPAD array combined with a TCSPC system (for lifetime measurement). These techniques lay on a wide-field illumination of the sample, without any scanning part. Conversely, FL-SMLM techniques do not require photon splitting for super-resolution lifetime imaging. In this section we will give an overview about recent progress in this direction, and in particular about FL-SMLM using rapid confocal-laser scanning fluorescence microscopes (CLSM) or novel wide-field detectors.

**Figure 10:**
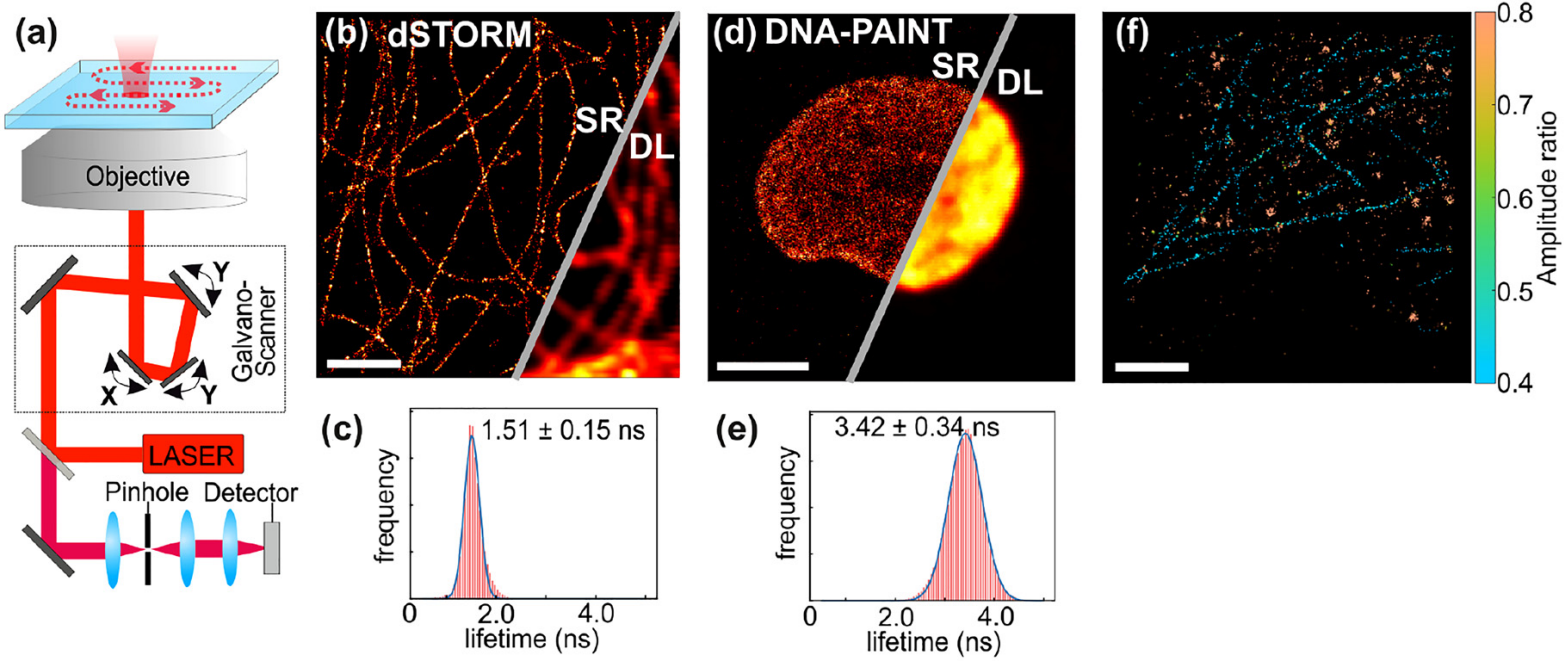
Confocal-laser scanning fluorescence microscopy (CLSM) for fluorescence-lifetime SMLM (FL-SMLM). (a) Schematic of the optical setup used for confocal SMLM, requiring a fast scanner, pulsed laser excitation, single-photon counting detector, and TCSPC electronics. (b) Confocal dSTORM image of tubulin filaments in human mesenchymal stem cells (hMSC) labeled with Alexa 647. Scale bar is 2 μm. (c) Lifetime histogram extracted from individual single-molecule localizations that constituted image (b). (d) Confocal DNA-PAINT image of chromatin in COS-7 cells. Histones H2B were labeled with Atto 655. Scale bar is 5 μm. Corresponding super-resolved (SR) and diffraction-limited (DL) images are shown both in (b) and (d). (e) Lifetime histogram for image shown in (d). (f) Multiplexed dSTORM image of fixed COS-7 cell. Tubulin is labeled with Alexa 647 and clathrin is labeled with Atto 655. Both targets were identified by their fluorescence lifetime using a pattern-matching algorithm. Scale bar is 2 μm. Adapted from Thiele et al. [187].

One of the most widely used techniques for FLIM for life science applications is based on CLSM equipped with time-correlated single photon counting (TCSPC). Such a system offers sufficient sensitivity for detecting single molecule, in contrast to phase-fluorometric systems that use sinusoidally time-modulated excitation together with phase-shifted time modulated wide-field detection. However, CLSM was never used for SMLM because of the relatively low overall light throughput of CLSM as compared with wide-field microscopy using cameras. Only recently, thanks to the employ of fast scanners which allow for recording images with frame rates close to video rates, it has been demonstrated that CLSM can also be used for SMLM. Together with suitable TCSPC electronics and single-photon counting detectors with high quantum efficiency of detection (e.g. silicon-based single-photon avalanche diodes), such systems allow for obtaining fluorescence-lifetime images with sufficient speed that can be then processed as in conventional wide-field SMLM [187], see also Figure 10. This made the realization of fluorescence lifetime dSTORM or DNA-PAINT possible, see Figure 10b–f for a few examples. One of the main benefits of the CLSM-based approach to FL-SMLM is the capability to take volumetric images due to the optical sectioning capability of CLSM, thus allowing for SMLM even deep inside tissues. In case of DNA-PAINT with CLSM, background signal from freely diffusing fluorophores in solution (unbound imager strands) is considerably reduced. The main disadvantage of CLSM-based FL-SMLM is the long acquisition time. This is due to relatively low light throughput of the technique: the same emitter has to be revisited by a scanning laser beam multiple times in order to collect a sufficient amount of photons for precise single-molecule localization. The total CLSM acquisition time is proportional to the size of a region of interest, making imaging of large regions (e.g. 20 μm × 20 μm or larger) extensively long.

**Figure 11:**
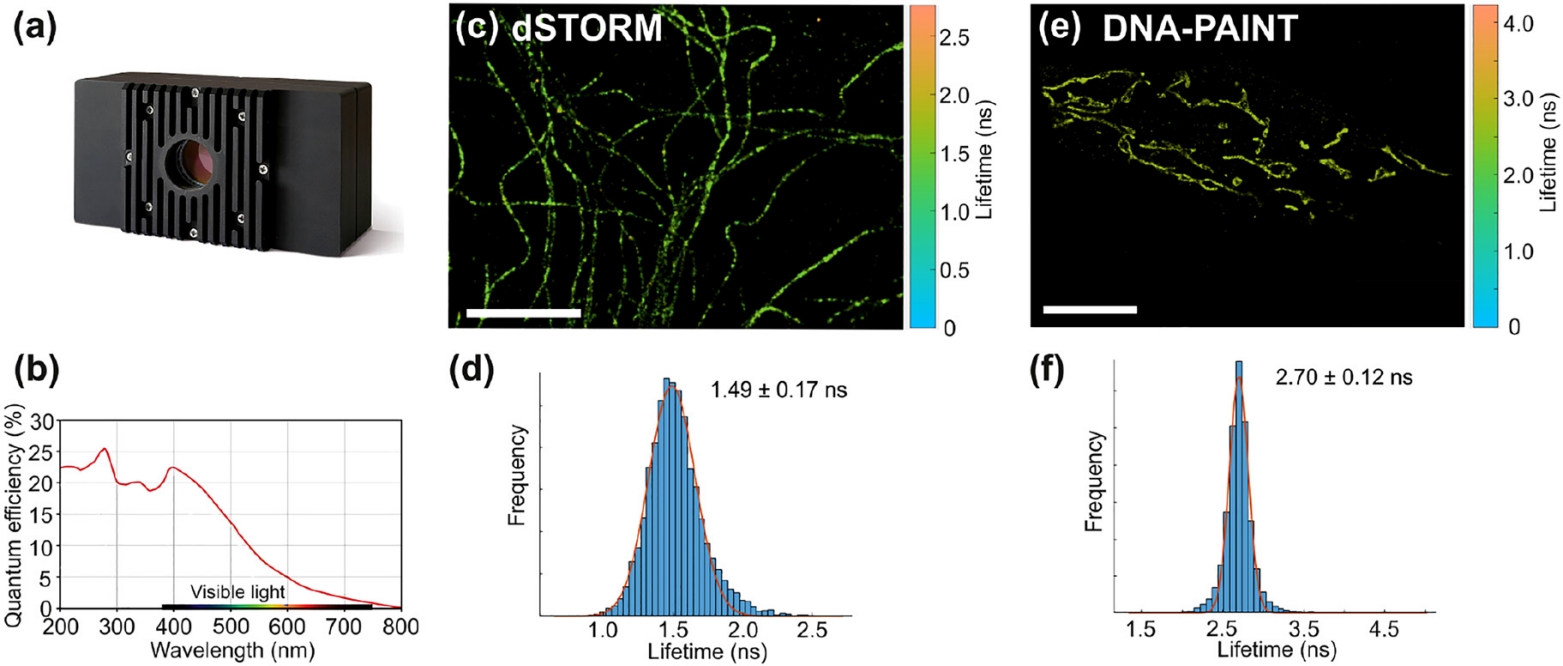
Fluorescence-lifetime SMLM using a wide-field imaging setup. (a) Commercially available TCSPC camera LINCam (Photonscore). (b) Quantum yield of the LINCam camera. (c) dSTORM FL-SMLM image of microtubules in hMSC labeled with Alexa 647. Scale bar is 5 μm. (d) Lifetime histogram extracted from individual single-molecule localizations in image (b). (e). DNA-PAINT FL-SMLM of mitochondria in HeLa cells labeled with Cy3b. Scale bar is 10 μm. Lifetime scale bars are shown on the right of images (c) and (e). (f). Lifetime histogram extracted from single-molecule localizations in image (e). Data was fitted with single Gaussian distributions. Average lifetime and standard deviation values are shown. The images in (a) and (b) are reprinted with permission of Photonscore GmbH.

In contrast to CLSM-based SMLM, wide-field SMLM has a higher throughput, enabling fast acquisition speed and the ability to image large fields of view. Moreover, wide-field SMLM can be easily combined with total internal reflection (TIR) excitation, or with highly inclined and laminated optical sheet (HILO) excitation. Up to date, only few technologies are available for wide-field FLIM with *single-molecule sensitivity*. Among them are time-gated SPAD arrays [133, 188], electron multiplying micro-channel plates (MCP) [189, 190], electro-optical modulators [191], or gated optical image intensifiers [192]. One of the most promising technologies for fluorescence-lifetime wide-field SMLM so far is the commercially available lifetime camera LINCam (Photonscore), see Figure 11a. It uses a micro-channel-plate photo-multiplier tube (MCP-PMT) and employs a capacity-coupled imaging technique combined with a charge division anode for accurate position readout [189]. The quantum yield (QY) of detection of this system has its maximum in the UV spectral region and drops from ∼20% in the blue to ∼5% in the red spectral regions, see Figure 11b. Despite this relatively low QY in the visible spectrum, the camera’s background and readout noise are almost completely absent, allowing even for single-molecule detection and localization with reasonable signal-to-noise ratio. Using the LINCam, single molecule imaging and localization has been successfully demonstrated even for the most challenging far-red spectral region [193], making the LINCam a perfect choice for wide-field FL-SMLM. As an example, ref. [193] demonstrated dSTORM imaging of microtubules in human mesenchymal stem cells (hMSC), see Figure 11c–d, and DNA-PAINT imaging of mitochondria in HeLa cells, see Figure 11e–f. Interestingly, the LINCam data output consists of an array of photon arrival times and coordinates, while the division into virtual pixels is done only after data acquisition. Therefore, pixel size in the final image can be adjusted and optimized by means of signal-to-noise ratio of a specific data set and pixelation artifacts can be reduced to a minimum.

Besides the obvious advantage of FL-SMLM for multiplexed imaging via the different lifetimes of different fluorophores, the most important advantage in the context of SMLM super-resolution is that FL-SMLM offers the possibility to do high-precision co-localization measurements between different fluorophores without any chromatic aberration artifacts. Conventional SMLM co-localization uses fluorophores of different colors, and although modern state-of-the art microscopy optical systems are well-corrected for their chromatic aberrations, remaining aberrations become increasingly noticeable when reaching spatial resolutions of a few nanometers. Here, FL-SMLM offers the ultimate solution because it allows for distinguishing between different but otherwise spectrally identical fluorophores solely on the basis of their different fluorescence lifetimes.

## 6 Conclusion and outlook

The past decade has seen the development of an exponentially increasing number of super-resolution localization microscopy techniques providing an unprecedented optical resolution in three-dimensions. Although the first applications of such techniques were mainly found in the life sciences, they specifically address issues that are common to the study of nanophotonic structures, such as time-resolved nanometer scale measurements of light–matter interaction at the single emitter level. Vice versa, the use of nanophotonic structures can find applications for studying biophysical problems. This review provided an overview of recent work in nanophotonics that uses fluorescence-based super-resolution microscopy techniques, with a particular emphasis on single-molecule localization microscopy, and of recent work in biophysics in which nanophotonic effects are employed. Sharing the knowledge between these two different and complementary fields has been demonstrated to have a valuable impact on both fields and will certainly be beneficial also in the future.

**Figure 12:**
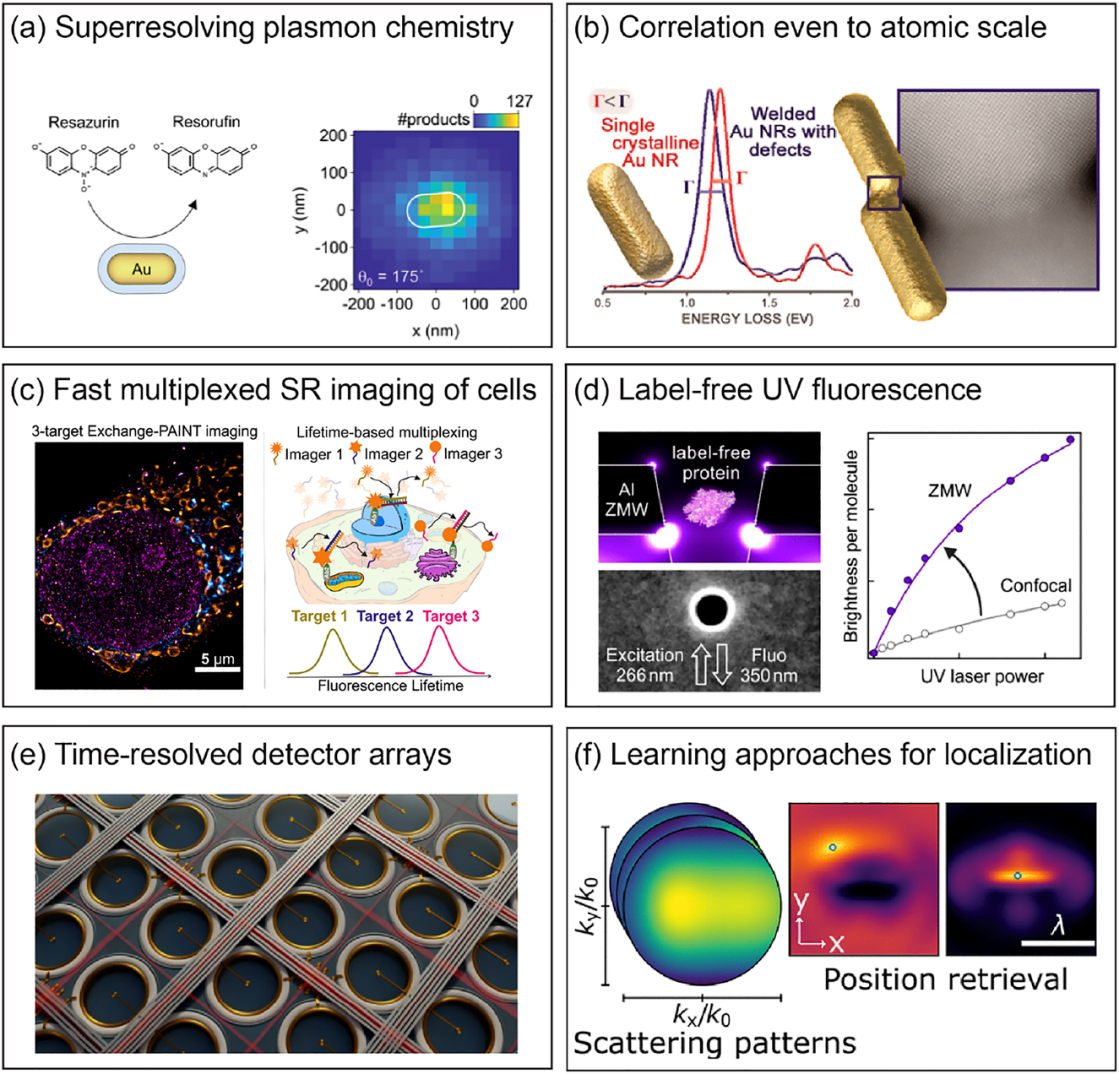
Outlook for SMLM in nanophotonics and biophysics. (a) Super-resolved plasmon chemistry at the single-molecule level (adapted from [194]). (b) Correlative microscopy at the atomic scale on gold nanorods (adapted from [195]). (c) Super-resolved *in cellulo* biochemistry (adapted from [83]). (d) Enhancing label-free UV fluorescence of proteins with ZWGs (adapted from [44]). (e) Artist view of a SPAD array (adapted from [196]). (f) Learning approaches to localization based on a library of diffraction patterns measured by Fourier microscopy (adapted from [113]).

As an outlook for the future, we identify a number of exciting opportunities that are summarized in Figure 12. First, an exciting new and actively discussed topic is combining plasmonics with chemistry (photochemistry): through a variety of mechanisms, plasmonic structures irradiated by light can boost the reaction rates of photochemical reactions. These mechanisms include hot electron chemistry, photocatalysis benefiting from confined light, thermal effects due to nanoscale heating, as well as reportedly quantum electrodynamic effects related to modifications of chemically relevant energy landscapes by strong light–matter interaction. Since each of these effects is strongly position dependent, deep insights can be gained from super-resolving chemical reaction sites on the surface of plasmonic systems. First important steps in this direction were taken by Hamans et al. [194] (see Figure 12a). Another exciting prospect in this field is to combine optical super-resolution microscopy with other microscopic techniques (see e.g. [195] and Figure 12b). For instance, LDOS control, SERS enhancements, nanoscale heating, or plasmon chemistry are increasingly studied in systems where atomic-scale geometric features are important. Recent developments in tomographic electron microscopy hint at the possibility to resolve the geometry of such nanoparticle-based photonic systems in three dimensions with elemental resolution. Correlating this with super-resolution localization microscopy of properties accessed by fluorophores could give unprecedentedly fine-grained insight into the relation between structure and function.

Analogously, a detailed understanding of the relation between structure and function at the single-molecule level is a fundamental ongoing quest in cell biology, with the added complexity of the highly dynamic and inhomogeneous nature of living cells. In order to decipher the molecular interplay underlying complex physiological processes, it is necessary to detect minute changes in biochemical states in response to physiological cues, ideally within their native cellular environment. As discussed in the previous section, advances in FLIM detectors do not only allow for imaging single molecules as required for super-resolution imaging, but do also provide fluorescence lifetime information that can be used for multiple purposes, for example for multiplexed imaging of biological objects (see Figure 12c). The main advantage of this approach is that multiple targets are imaged simultaneously, resulting in faster image acquisition of multiple targets as compared with conventional sequential imaging as e.g. used in Exchange-PAINT [83]. Another fascinating avenue is that new SMLM approaches such as PAINT or MINFLUX, potentially in combination with nanophotonic methods such as MIET or GIET, are coming closer to the dream of nanometric three-dimensional resolution, so that they could be used for structural biology of individual biomolecules or biomolecular complexes, complemented the much more challenging methods from X-ray scattering and cryo-electron microscopy.

Another challenge in bioimaging is the quest for label-free detection of proteins, allowing an unperturbed observation of molecular structure and function. When considering protein or DNA autofluorescence, the main challenge here is to achieve a sufficiently high detection sensitivity in the UV, where protein or DNA autofluorescence occurs. The combination of expertise from biophysics and nanophotonics turned out to be particularly fruitful for addressing this challenge. Indeed, detecting the very weak signal coming from protein UV autofluorescence was recently demonstrated thanks to the use of aluminum ZWGs [44] (see Figure 12d). The interaction of a single protein with the ZWG enhances protein autofluorescence and enables detection of proteins at physiological concentrations. Optimized nanophotonics systems will improve fluorescence enhancement, eventually allowing for single-molecule biophysical studies of large protein libraries.

Further technological innovations can also be expected to improve super-resolution localization microscopy and its applications to studying nanophotonic structures or biological systems. Detector development for SMLM has to face multiple challenges, such as high quantum efficiency across the whole visible spectrum, low noise, high temporal resolution, large number of pixels and, last but not least, easy data handling. Among the variety of single-photon detectors with time resolution capability, such as Photo-Multiplier Tubes (PMTs), Micro-Channel Plates (MCPs), Single-Photon Avalanche Diodes (SPADs), or streak cameras, the most promising ones, which are undergoing a fast optimization of their specifications, seem to be SPAD arrays and MCP-based cameras [197]. In the present review, we have discussed applications of a recently development MCP-based camera (LINCam) and of SPAD arrays for super-resolution lifetime imaging. CMOS-based custom-built SPAD arrays represent an emerging class of imaging detectors [196, 198] offering megapixel detector size, high sensitivity, and high temporal resolution (see Figure 12e). In parallel, in the field of high-energy physics, pixelated time-resolved microchip detector technology has been developed, such as the Medipix/Timepix family of detectors, providing also nanosecond temporal resolution.

Aside from new developments in hardware, we do also expect improvements in algorithms that can boost localization. The fact that the PSF and the radiation pattern are strongly modified for emitters close to nanophotonic structures can be used as a resource for improving the localization precision and accuracy, since these modifications depend on the relative position of an emitter with respect to the nanostructure. While it is a Herculean task to perform physics-based simulations of such experiments, data-driven techniques that are based on *a priori* measured calibration libraries of the PSF and radiation pattern as function of the geometry may provide new ways of efficient data analysis that are computationally affordable. For instance, Buijs et al. [113] (see Figure 12f) recently reported a *λ*/200 localization precision of an emitter with respect to a nanostructure by using just a library of diffraction patterns.

In summary, the combination of ideas, tool, and concepts from super-resolution bioimaging with those from nanophotonics are highly fruitful and inspiring for both fields of research: cutting-edge SMLM helps to gain deeper insight into fundamental light–matter interaction in nanophotonic devices and structures, while physical principles and effects from nanophotonics and plasmonics help to devise new methods of super-resolution microscopy that have unprecedented accuracy and precision. We hope that our review could give a fair overview of the current state-of-the art of the extremely prolific interplay between super-resolution SMLM and nanophotonics, and that it will inspire future new developments at the interface of both fields.

## References

[1] Sandoghdar V. (2020). Nano-optics in 2020 ± 20. Nano Lett..

[2] Koenderink A. F., Alu A., Polman A. (2015). Nanophotonics: shrinking light-based technology. Science.

[3] Thomson D., Zilkie A., Bowers J. E. (2016). Roadmap on silicon photonics. J. Opt..

[4] Vahala K. J. (2003). Optical microcavities. Nature.

[5] Oh S. H., Altug H., Jin X. (2021). Nanophotonic biosensors harnessing van der Waals materials. Nat. Commun..

[6] Zhang S., Wong C. L., Zeng S. (2021). Metasurfaces for biomedical applications: imaging and sensing from a nanophotonics perspective. Nanophotonics.

[7] Cortés E., Besteiro L. V., Alabastri A. (2020). Challenges in plasmonic catalysis. ACS Nano.

[8] Baffou G., Quidant R. (2014). Nanoplasmonics for chemistry. Chem. Soc. Rev..

[9] Lozano G., Rodriguez S. R., Verschuuren M. A., Rivas J. G. (2016). Metallic nanostructures for efficient LED lighting. Light Sci. Appl..

[10] Raimond J. M., Brune M., Haroche S. (2001). Manipulating quantum entanglement with atoms and photons in a cavity. Rev. Mod. Phys..

[11] Santori C., Fattal D., Yamamoto Y. (2010). Single-photon Devices and Applications.

[12] Lodahl P., Mahmoodian S., Stobbe S. (2015). Interfacing single photons and single quantum dots with photonic nanostructures. Rev. Mod. Phys..

[13] Senellart P., Solomon G., White A. (2017). High-performance semiconductor quantum-dot single-photon sources. Nat. Nanotechnol..

[14] Arcari M., Söllner I., Javadi A. (2014). Near-unity coupling efficiency of a quantum emitter to a photonic crystal waveguide. Phys. Rev. Lett..

[15] Aharonovich I., Neu E. (2014). Diamond nanophotonics. Adv. Opt. Mater..

[16] Low T., Chaves A., Caldwell J. (2017). Polaritons in layered two-dimensional materials. *Nat. Mater.*.

[17] Maier S. (2007). *Plasmonics: Fundamentals and Applications*.

[18] Bharadwaj P., Deutsch B., Novotny L. (2009). Optical antennas. Adv. Opt. Photon.

[19] Novotny L., Van Hulst N. (2011). Antennas for light. Nat. Photonics.

[20] Koenderink A. F. (2017). Single-photon nano-antennas. ACS Photonics.

[21] Akselrod G. M., Argyropoulos C., Hoang T. B. (2014). Probing the mechanisms of large Purcell enhancement in plasmonic nanoantennas. Nat. Photonics.

[22] Chikkaraddy R., de Nijs B., Benz F. (2016). Single-molecule strong coupling at room temperature in plasmonic nanocavities. Nature.

[23] Bergman D. J., Stockman M. I. (2003). Surface plasmon amplification by stimulated emission of radiation: quantum generation of coherent surface plasmons in nanosystems. Phys. Rev. Lett..

[24] Wang W., Ramezani M., Väkeväinen A. I., Törmä P., Rivas J. G., Odom T. W. (2018). The rich photonic world of plasmonic nanoparticle arrays. Mater. Today.

[25] Benz F., Schmidt M., Dreismann A. (2016). Single-molecule optomechanics in picocavities. Science.

[26] Roelli P., Galland C., Piro N., Kippenberg T. (2016). Molecular cavity optomechanics as a theory of plasmon-enhanced Raman scattering. Nat. Nanotechnol..

[27] Trofymchuk K., Glembockyte V., Grabenhorst L. (2021). Addressable nanoantennas with cleared hotspots for single-molecule detection on a portable smartphone microscope. Nat. Commun..

[28] Ebbesen T. W., Lezec H. J., Ghaemi H. F., Thio T., Wolff P. A. (1998). Extraordinary optical transmission through sub-wavelength hole arrays. Nature.

[29] Samiee K. T., Moran-Mirabal J. M., Cheung Y. K., Craighead H. G. (2006). Zero mode waveguides for single-molecule spectroscopy on lipid membranes. Biophys. J..

[30] Wenger J., Rigneault H., Dintinger J., Marguet D., Lenne P. F. (2006). Single-fluorophore diffusion in a lipid membrane over a subwavelength aperture. J. Biol. Phys..

[31] Wenger J., Conchonaud F., Dintinger J. (2007). Diffusion analysis within single nanometric apertures reveals the ultrafine cell membrane organization. Biophys. J..

[32] Flauraud V., Regmi R., Winkler P. M. (2017). In-plane plasmonic antenna arrays with surface nanogaps for giant fluorescence enhancement. Nano Lett..

[33] Winkler P. M., Regmi R., Flauraud V. (2017). Transient nanoscopic phase separation in biological lipid membranes resolved by planar plasmonic antennas. ACS Nano.

[34] Regmi R., Winkler P. M., Flauraud V. (2017). Planar optical nanoantennas resolve cholesterol-dependent nanoscale heterogeneities in the plasma membrane of living cells. Nano Lett..

[35] Winkler P. M., Regmi R., Flauraud V. (2018). Optical antenna-based fluorescence correlation spectroscopy to probe the nanoscale dynamics of biological membranes. J. Phys. Chem. Lett..

[36] Pradhan B., Khatua S., Gupta A., Aartsma T., Canters G., Orrit M. (2016). Gold-nanorod-enhanced fluorescence correlation spectroscopy of fluorophores with high quantum yield in lipid bilayers. J. Phys. Chem. C.

[37] Eid J., Fehr A., Gray J. (2009). Real-time DNA sequencing from single polymerase molecules. Science.

[38] Flusberg B. A., Webster D. R., Lee J. H. (2010). Direct detection of DNA methylation during single-molecule, real-time sequencing. Nat. Methods.

[39] Ghenuche P., de Torres J., Moparthi S. B., Grigoriev V., Wenger J. (2014). Nanophotonic enhancement of the förster resonance energy-transfer rate with single nanoapertures. Nano Lett..

[40] Ghenuche P., Mivelle M., de Torres J. (2015). Matching nanoantenna field confinement to FRET distances enhances förster energy transfer rates. Nano Lett..

[41] Bidault S., Devilez A., Ghenuche P., Stout B., Bonod N., Wenger J. (2016). Competition between förster resonance energy transfer and donor photodynamics in plasmonic dimer nanoantennas. ACS Photonics.

[42] Baibakov M., Patra S., Claude J. B., Moreau A., Lumeau J., Wenger J. (2019). Extending single-molecule förster resonance energy transfer (FRET) range beyond 10 nanometers in zero-mode waveguides. ACS Nano.

[43] de Torres J., Mivelle M., Moparthi S. B. (2016). Plasmonic nanoantennas enable forbidden förster dipole–dipole energy transfer and enhance the FRET efficiency. Nano Lett..

[44] Barulin A., Wenger J. (2020). Ultraviolet photostability improvement for autofluorescence correlation spectroscopy on label-free proteins. J. Phys. Chem. Lett..

[45] Huang B., Bates M., Zhuang X. (2009). Super-resolution fluorescence microscopy. Annu. Rev. Biochem..

[46] Hell S. W. (2007). Far-field optical nanoscopy. Science.

[47] Hell S. W., Wichmann J. (1994). Breaking the diffraction resolution limit by stimulated emission: stimulated-emission-depletion fluorescence microscopy. Opt. Lett..

[48] Klar T. A., Jakobs S., Dyba M., Egner A., Hell S. W. (2000). Fluorescence microscopy with diffraction resolution barrier broken by stimulated emission. Proc. Natl. Acad. Sci. U. S. A..

[49] Fölling J., Bossi M., Bock H. (2008). Fluorescence nanoscopy by ground-state depletion and single-molecule return. Nat. Methods.

[50] Hell S. W. (2009). Microscopy and its focal switch. Nat. Methods.

[51] Schwentker A. M., Bock H., Hofmann M. (2007). Wide-field subdiffraction RESOLFT microscopy using fluorescent protein photoswitching. Microsc. Res. Tech..

[52] Keller J., Schönle A., Hell S. W. (2007). Efficient fluorescence inhibition patterns for RESOLFT microscopy. Opt. Express.

[53] Heintzmann R., Jovin T. M., Cremer C. (2002). Saturated patterned excitation microscopy—a concept for optical resolution improvement. JOSA A.

[54] Gustafsson M. G. L. (2005). Nonlinear structured-illumination microscopy: wide-field fluorescence imaging with theoretically unlimited resolution. Proc. Natl. Acad. Sci. U.S.A..

[55] Humpolíčková J., Benda A., Enderlein J. (2009). Optical saturation as a versatile tool to enhance resolution in confocal microscopy. Biophys. J..

[56] Fujita K., Kobayashi M., Kawano S., Yamanaka M., Kawata S. (2007). High-resolution confocal microscopy by saturated excitation of fluorescence. Phys. Rev. Lett..

[57] Orrit M., Bernard J. (1990). Single pentacene molecules detected by fluorescence excitation in a p-terphenyl crystal. Phys. Rev. Lett..

[58] Moerner W. E., Orrit M. (1999). Illuminating single molecules in condensed matter. Science.

[59] Klein T., Proppert S., Sauer M. (2014). Eight years of single-molecule localization microscopy. Histochem. Cell Biol..

[60] Betzig E., Patterson G. H., Sougrat R. (2006). Imaging intracellular fluorescent proteins at nanometer resolution. Science.

[61] Hess S. T., Gould T. J., Gunewardene M., Bewersdorf J., Mason M. D. (2009). Ultrahigh resolution imaging of biomolecules by fluorescence photoactivation localization microscopy. *Micro and Nano Technologies in Bioanalysis*.

[62] Rust M. J., Bates M., Zhuang X. (2006). Sub-diffraction-limit imaging by stochastic optical reconstruction microscopy (STORM). Nat. Methods.

[63] Van de Linde S., Löschberger A., Klein T. (2011). Direct stochastic optical reconstruction microscopy with standard fluorescent probes. Nat. Protoc..

[64] Sharonov A., Hochstrasser R. M. (2006). Wide-field subdiffraction imaging by accumulated binding of diffusing probes. Proc. Natl. Acad. Sci. U. S. A..

[65] Balzarotti F., Eilers Y., Gwosch K. C. (2017). Nanometer resolution imaging and tracking of fluorescent molecules with minimal photon fluxes. Science.

[66] Abbe E. (1873). Beiträge zur Theorie des Mikroskops und der mikroskopischen Wahrnehmung. Arch. Mikrosk. Anat..

[67] Ober R. J., Ram S., Ward E. S. (2004). Localization accuracy in single-molecule microscopy. Biophys. J..

[68] Enderlein J., Toprak E., Selvin P. R. (2006). Polarization effect on position accuracy of fluorophore localization. Opt. Express.

[69] Engelhardt J., Keller J., Hoyer P., Reuss M., Staudt T., Hell S. W. (2011). Molecular orientation affects localization accuracy in superresolution far-field fluorescence microscopy. Nano Lett..

[70] Lieb M. A., Zavislan J. M., Novotny L. (2004). Single-molecule orientations determined by direct emission pattern imaging. J. Opt. Soc. Am. B.

[71] Patra D., Gregor I., Enderlein J. (2004). Image analysis of defocused single-molecule images for three-dimensional molecule orientation studies. J. Phys. Chem..

[72] Kurvits J. A., Jiang M., Zia R. (2015). Comparative analysis of imaging configurations and objectives for Fourier microscopy. J. Opt. Soc. Am. A.

[73] Backer A. S., Moerner W. E. (2014). Extending single-molecule microscopy using optical fourier processing. J. Phys. Chem. B.

[74] Curcio V., Alemán-Castañeda L. A., Brown T. G., Brasselet S., Alonso M. A. (2020). Birefringent Fourier filtering for single molecule coordinate and height super-resolution imaging with dithering and orientation. *Nat. Commun.*.

[75] Cruz C. A. V., Shaban H. A., Kress A. (2016). Quantitative nanoscale imaging of orientational order in biological filaments by polarized superresolution microscopy. Proc. Natl. Acad. Sci. U.S.A..

[76] Nevskyi O., Tsukanov R., Gregor I., Karedla N., Enderlein J. (2020). Fluorescence polarization filtering for accurate single molecule localization. APL Photonics.

[77] Lavis L. D. (2017). Chemistry is dead. Long live chemistry. Biochemistry.

[78] Jungmann R., Steinhauer C., Scheible M., Kuzyk A., Tinnefeld P., Simmel F. C. (2010). Single-molecule kinetics and super-resolution microscopy by fluorescence imaging of transient binding on DNA origami. Nano Lett..

[79] Schnitzbauer J., Strauss M. T., Schlichthaerle T., Schueder F., Jungmann R. (2017). Super-resolution microscopy with DNA-PAINT. Nat. Protoc..

[80] Auer A., Schlichthaerle T., Woehrstein J. B. (2018). Nanometer-scale multiplexed super-resolution imaging with an economic 3D-DNA-PAINT microscope. ChemPhysChem.

[81] Wade O. K., Woehrstein J. B., Nickels P. C. (2019). 124-Color super-resolution imaging by engineering DNA-PAINT blinking kinetics. Nano Lett..

[82] Jungmann R., Avendaño M. S., Woehrstein J. B., Dai M., Shih W. M., Yin P. (2014). Multiplexed 3D cellular super-resolution imaging with DNA-PAINT and Exchange-PAINT. Nat. Methods.

[83] Sograte-Idrissi S., Oleksiievets N., Isbaner S. (2019). Nanobody detection of standard fluorescent proteins enables multi-target DNA-PAINT with high resolution and minimal displacement errors. Cells.

[84] Eilers Y., Ta H., Gwosch K. C., Balzarotti F., Hell S. W. (2018). MINFLUX monitors rapid molecular jumps with superior spatiotemporal resolution. Proc. Natl. Acad. Sci. U. S. A..

[85] Nieuwenhuizen R. P. J., Lidke K. A., Bates M. (2013). Measuring image resolution in optical nanoscopy. Nat. Methods.

[86] Salas D., Gall A. L., Fiche J. B. (2017). Angular reconstitution-based 3D reconstructions of nanomolecular structures from superresolution light-microscopy images. Proc. Natl. Acad. Sci. Unit. States Am..

[87] Bouchet D., Krachmalnicoff V., Izeddin I. (2019). Cramér-Rao analysis of lifetime estimations in time-resolved fluorescence microscopy. Opt. Express.

[88] Yang J., Hugonin J. P., Lalanne P. (2016). Near-to-Far field transformations for radiative and guided waves. ACS Photonics.

[89] Anger P., Bharadwaj P., Novotny L. (2006). Enhancement and quenching of single-molecule fluorescence. Phys. Rev. Lett..

[90] Kühn S., Håkanson U., Rogobete L., Sandoghdar V. (2006). Enhancement of single-molecule fluorescence using a gold nanoparticle as an optical nanoantenna. Phys. Rev. Lett..

[91] Matsuzaki K., Liu H. W., Götzinger S., Sandoghdar V. (2021). On quantum efficiency measurements and plasmonic antennas. ACS Photonics.

[92] Drexhage K., Fleck M., Shäfer F., Sperling W. (1966). Beeinflussung der Fluoreszenz eines Europium-chelates durch einen Spiegel. Ber. Bunsenges. Phys. Chem..

[93] Snoeks E., Lagendijk A., Polman A. (1995). Measuring and modifying the spontaneous emission rate of erbium near an interface. Phys. Rev. Lett..

[94] Amos R. M., Barnes W. L. (1997). Modification of the spontaneous emission rate of Eu3+ ions close to a thin metal mirror. Phys. Rev. B.

[95] Brokmann X., Coolen L., Dahan M., Hermier J. (2004). Measurement of the radiative and nonradiative decay rates of single CdSe nanocrystals through a controlled modification of their spontaneous emission. Phys. Rev. Lett..

[96] Buchler B. C., Kalkbrenner T., Hettich C., Sandoghdar V. (2005). Measuring the quantum efficiency of the optical emission of single radiating dipoles using a scanning mirror. Phys. Rev. Lett..

[97] Leistikow M., Johansen J., Kettelarij A., Lodahl P., Vos W. (2009). Size-dependent oscillator strength and quantum efficiency of CdSe quantum dots controlled via the local density of states. Phys. Rev. B.

[98] Chizhik A. I., Chizhik A. M., Khoptyar D., Ba S., Meixner A. J., Enderlein J. (2011). Probing the radiative transition of single molecules with a tunable microresonator. Nano Lett..

[99] Frimmer M., Mohtashami A., Femius Koenderink A. (2013). Nanomechanical method to gauge emission quantum yield applied to nitrogen-vacancy centers in nanodiamond. Appl. Phys. Lett..

[100] Lunnemann P., Rabouw F. T., van Dijk-Moes R. J. A., Pietra F., Vanmaekelbergh D., Koenderink A. F. (2013). Calibrating and controlling the quantum efficiency distribution of inhomogeneously broadened quantum rods by using a mirror ball. ACS Nano.

[101] Chizhik A. I., Gregor I., Ernst B., Enderlein J. (2013). Nanocavity-based determination of absolute values of photoluminescence quantum yields. ChemPhysChem.

[102] Chizhik A. I., Gregor I., Enderlein J. (2013). Quantum yield measurement in a multicolor chromophore solution using a nanocavity. Nano Lett..

[103] Barnes W. L., Horsley S. A. R., Vos W. L. (2020). Classical antennae, quantum emitters, and densities of optical states. *J. Opt.*.

[104] Sersic I., Tuambilangana C., Koenderink A. F. (2011). Fourier microscopy of single plasmonic scatterers. New J. Phys..

[105] Röhrich R., Hoekmeijer C., Osorio C. I., Koenderink A. F. (2018). Quantitative single nano-antenna far fields through interferometric and polarimetric k-space microscopy. Light Sci. Appl..

[106] Kinkhabwala A., Yu Z., Fan S., Avlasevich Y., Muellen K., Moerner W. E. (2009). Large single-molecule fluorescence enhancements produced by a bowtie nanoantenna. Nat. Photonics.

[107] Carminati R., Cazé A., Cao D. (2015). Electromagnetic density of states in complex plasmonic systems. Surf. Sci. Rep..

[108] Purcell E. M. (1946). Spontaneous emission probabilities at radio frequencies. Phys. Rev..

[109] Aouani H., Mahboub O., Devaux E., Rigneault H., Ebbesen T. W., Wenger J. (2011). Plasmonic antennas for directional sorting of fluorescence emission. Nano Lett..

[110] Langguth L., Punj D., Wenger J., Koenderink A. F. (2013). Plasmonic band structure controls single-molecule fluorescence. ACS Nano.

[111] van der Burgt J. S., Garnett E. C. (2020). Nanophotonic emission control for improved photovoltaic efficiency. ACS Photonics.

[112] Vaskin A., Kolkowski R., Koenderink A. F., Staude I. (2019). Light emitting metasurfaces. Nanophotonics.

[113] Buijs R. D., Schilder N. J., Wolterink T. A. W., Gerini G., Verhagen E., Koenderink A. F. (2020). Super-resolution without imaging: library-based approaches using near-to-far-field transduction by a nanophotonic structure. ACS Photonics.

[114] Sandoghdar V. (2005). Trends and developments in scanning near-field optical microscopy. Proc. Int. Sch. Phys. Enrico Fermi.

[115] Kühn S., Hettich C., Schmitt C., Poizat J. P., Sandoghdar V. (2001). Diamond colour centres as a nanoscopic light source for scanning near-field optical microscopy. J Microsc.

[116] Stranahan S. M., Willets K. A. (2010). Super-resolution optical imaging of single-molecule SERS hot spots. Nano Lett..

[117] Cang H., Labno A., Lu C. (2011). Probing the electromagnetic field of a 15-nanometre hotspot by single molecule imaging. Nature.

[118] Weber M. L., Willets K. A. (2011). Correlated super-resolution optical and structural studies of surface-enhanced Raman scattering hot spots in silver colloid aggregates. J. Phys. Chem. Lett..

[119] Weber M. L., Litz J. P., Masiello D. J., Willets K. A. (2012). Super-resolution imaging reveals a difference between SERS and luminescence centroids. ACS Nano.

[120] Willets K. A., Stranahan S. M., Weber M. L. (2012). Shedding light on surface-enhanced Raman scattering hot spots through single-molecule super-resolution imaging. J. Phys. Chem. Lett..

[121] Su L., Yuan H., Lu G. (2016). Super-resolution localization and defocused fluorescence microscopy on resonantly coupled single-molecule, single-nanorod hybrids. ACS Nano.

[122] Wertz E. A., Isaacoff B. P., Biteen J. S. (2016). Wavelength-dependent super-resolution images of dye molecules coupled to plasmonic nanotriangles. ACS Photonics.

[123] Mack D. L., Cortés E., Giannini V., Török P., Roschuk T., Maier S. A. (2017). Decoupling absorption and emission processes in super-resolution localization of emitters in a plasmonic hotspot. Nat. Commun..

[124] Johlin E., Solari J., Mann S. A., Wang J., Shimizu T. S., Garnett E. C. (2016). Super-resolution imaging of light–matter interactions near single semiconductor nanowires. Nat. Commun..

[125] Raab M., Vietz C., Stefani F. D., Acuna G. P., Tinnefeld P. (2017). Shifting molecular localization by plasmonic coupling in a single-molecule mirage. Nat. Commun..

[126] Wertz E., Isaacoff B. P., Flynn J. D., Biteen J. S. (2015). Single-molecule super-resolution microscopy reveals how light couples to a plasmonic nanoantenna on the nanometer scale. Nano Lett..

[127] Ropp C., Cummins Z., Nah S., Fourkas J. T., Shapiro B., Waks E. (2015). Nanoscale probing of image-dipole interactions in a metallic nanostructure. Nat. Commun..

[128] Hamans R. F., Parente M., Castellanos G. W., Ramezani M., Gómez Rivas J., Baldi A. (2019). Super-resolution mapping of enhanced emission by collective plasmonic resonances. ACS Nano.

[129] Groß H., Heil H. S., Ehrig J., Schwarz F. W., Hecht B., Diez S. (2018). Parallel mapping of optical near-field interactions by molecular motor-driven quantum dots. Nat. Nanotechnol..

[130] Ropp C., Cummins Z., Nah S., Fourkas J., Shapiro B., Waks E. (2013). Nanoscale imaging and spontaneous emission control with a single nano-positioned quantum dot. Nat. Commun..

[131] Guo K., Verschuuren M. A., Koenderink A. F. (2016). Superresolution imaging of the local density of states in plasmon lattices. Optica.

[132] Bouchet D., Scholler J., Blanquer G., Wilde Y. D., Izeddin I., Krachmalnicoff V. (2019). Probing near-field light-matter interactions with single-molecule lifetime imaging. Optica.

[133] Blanquer G., van Dam B., Gulinatti A. (2020). Relocating single molecules in super-resolved fluorescence lifetime images near a plasmonic nanostructure. ACS Photonics.

[134] Willets K. A. (2014). Plasmon point spread functions: how do we model plasmon-mediated emission processes?. Front. Physiol..

[135] Willets K. A. (2014). Super-resolution imaging of SERS hot spots. Chem. Soc. Rev..

[136] Lim K., Ropp C., Barik S., Fourkas J., Shapiro B., Waks E. (2016). Nanostructure-induced distortion in single-emitter microscopy. Nano Lett..

[137] Willets K. A., Wilson A. J., Sundaresan V., Joshi P. B. (2017). Super-resolution imaging and plasmonics. Chem. Rev..

[138] Su L., Lu G., Kenens B. (2015). Visualization of molecular fluorescence point spread functions via remote excitation switching fluorescence microscopy. Nat. Commun..

[139] Baiyasi R., Jebeli S. A. H., Zhang Q. (2019). PSF distortion in dye–plasmonic nanomaterial interactions: friend or foe?. ACS Photonics.

[140] Xin L., Lu M., Both S. (2019). Watching a single fluorophore molecule walk into a plasmonic hotspot. ACS Photonics.

[141] Lalanne P., Yan W., Vynck K., Sauvan C., Hugonin J. P. (2018). Light interaction with photonic and plasmonic resonances. Laser Photon. Rev..

[142] Shegai T., Brian B., Miljković V. D., Käll M. (2011). Angular distribution of surface-enhanced Raman scattering from individual Au nanoparticle aggregates. ACS Nano.

[143] Löschberger A., van de Linde S., Dabauvalle M. C. (2012). Super-resolution imaging visualizes the eightfold symmetry of gp210 proteins around the nuclear pore complex and resolves the central channel with nanometer resolution. J. Cell Sci..

[144] Willig K. I., Harke B., Medda R., Hell S. W. (2007). STED microscopy with continuous wave beams. Nat. Methods.

[145] Zhang W., Noa A., Nienhaus K., Hilbert L., Nienhaus G. U. (2019). Super-resolution imaging of densely packed DNA in nuclei of zebrafish embryos using stimulated emission double depletion microscopy. J. Phys. D Appl. Phys..

[146] Huang B., Wang W., Bates M., Zhuang X. (2008). Three-dimensional super-resolution imaging by stochastic optical reconstruction microscopy. Science.

[147] Babcock H., Sigal Y. M., Zhuang X. (2012). A high-density 3D localization algorithm for stochastic optical reconstruction microscopy. Opt. Nanoscopy.

[148] Juette M. F., Gould T. J., Lessard M. D. (2008). Three-dimensional sub–100nmresolution fluorescence microscopy of thick samples. Nat. Methods.

[149] Pavani S. R. P., Thompson M. A., Biteen J. S. (2009). Three-dimensional, single-molecule fluorescence imaging beyond the diffraction limit by using a double-helix point spread function. Proc. Natl. Acad. Sci. U. S. A..

[150] Shechtman Y., Weiss L. E., Backer A. S., Sahl S. J., Moerner W. (2015). Precise three-dimensional scan-free multiple-particle tracking over large axial ranges with tetrapod point spread functions. Nano Lett..

[151] Bon P., Bourg N., Lécart S. (2015). Three-dimensional nanometre localization of nanoparticles to enhance super-resolution microscopy. Nat. Commun..

[152] Bon P., Linarès-Loyez J., Feyeux M., Alessandri K., Lounis B., Cognet L. (2018). Self-interference 3D super-resolution microscopy for deep tissue investigations. Nat. Methods.

[153] Shtengel G., Galbraith J. A., Galbraith C. G. (2009). Interferometric fluorescent super-resolution microscopy resolves 3D cellular ultrastructure. Proc. Natl. Acad. Sci. U. S. A..

[154] Schmidt R., Wurm C. A., Jakobs S., Engelhardt J., Egner A., Hell S. W. (2008). Spherical nanosized focal spot unravels the interior of cells. Nat. Methods.

[155] Jouchet P., Cabriel C., Bourg N. (2021). Nanometric axial localization of single fluorescent molecules with modulated excitation. Nat. Photonics.

[156] Gwosch K. C., Pape J. K., Balzarotti F. (2020). MINFLUX nanoscopy delivers 3D multicolor nanometer resolution in cells. Nat. Methods.

[157] Burmeister J. S., Truskey G. A., Reichert W. M. (1994). Quantitative analysis of variable-angle total internal reflection fluorescence microscopy (VA-TIRFM) of cell/substrate contacts. J. Microsc..

[158] Stock K., Sailer R., Strauss W. S., Lyttek M., Steiner R., Schneckenburger H. (2003). Variable-angle total internal reflection fluorescence microscopy (VA-TIRFM): realization and application of a compact illumination device. J Microsc.

[159] Dos Santos M. C., Déturche R., Vézy C., Jaffiol R. (2016). Topography of cells revealed by variable-angle total internal reflection fluorescence microscopy. Biophys. J..

[160] Ruckstuhl T., Verdes D. (2004). Supercritical angle fluorescence (SAF) microscopy. Opt. Express.

[161] Winterflood C. M., Ruckstuhl T., Verdes D., Seeger S. (2010). Nanometer axial resolution by three-dimensional supercritical angle fluorescence microscopy. Phys. Rev. Lett..

[162] Deschamps J., Mund M., Ries J. (2014). 3D superresolution microscopy by supercritical angle detection. Opt. Express.

[163] Bourg N., Mayet C., Dupuis G. (2015). Direct optical nanoscopy with axially localized detection. Nat. Photonics.

[164] Chizhik A. I., Rother J., Gregor I., Janshoff A., Enderlein J. (2014). Metal-induced energy transfer for live cell nanoscopy. Nat. Photonics.

[165] Lukosz W., Kunz R. (1977). Light emission by magnetic and electric dipoles close to a plane interface. I. Total radiated power. J. Opt. Soc. Am. A.

[166] Lukosz W., Kunz R. (1977). Fluorescence lifetime of magnetic and electric dipoles near a dielectric interface. Opt. Commun..

[167] Chance R., Prock A., Silbey R. (1978). Molecular fluorescence and energy transfer near interfaces. Adv. Chem. Phys..

[168] Drexhage K., Kuhn H., Schäfer F. (1968). Variation of the fluorescence decay time of a molecule in front of a mirror. Ber. Bunsenges. Phys. Chem..

[169] Tews K. H., Inacker O., Kuhn H. (1970). Variation of the luminescence lifetime of a molecule near an interface between differently polarizable dielectrics. Nature.

[170] Chizhik A. M., Wollnik C., Ruhlandt D. (2018). Dual-color metal-induced and Förster resonance energy transfer for cell nanoscopy. Mol. Biol. Cell.

[171] Baronsky T., Ruhlandt D., Brückner B. R. (2017). Cell–substrate dynamics of the epithelial-to-mesenchymal transition. Nano Lett..

[172] Chizhik A. M., Ruhlandt D., Pfaff J. (2017). Three-dimensional reconstruction of nuclear envelope architecture using dual-color metal-induced energy transfer imaging. ACS Nano.

[173] Figueiras E., Silvestre O. F., Ihalainen T. O., Nieder J. B. (2019). Phasor-assisted nanoscopy reveals differences in the spatial organization of major nuclear lamina proteins. Biochim. Biophys. Acta Mol. Cell Res..

[174] Hwang W., Seo J., Kim D. (2021). Large field-of-view nanometer-sectioning microscopy by using metal-induced energy transfer and biexponential lifetime analysis. Commun. Biol..

[175] Karedla N., Chizhik A. I., Gregor I., Chizhik A. M., Schulz O., Enderlein J. (2014). Single-molecule metal-induced energy transfer (smMIET): resolving nanometer distances at the single-molecule level. ChemPhysChem.

[176] Isbaner S., Karedla N., Kaminska I. (2018). Axial colocalization of single molecules with nanometer accuracy using metal-induced energy transfer. Nano Lett..

[177] Kapusta P., Wahl M., Benda A., Hof M., Enderlein J. (2007). Fluorescence lifetime correlation spectroscopy. J. Fluoresc..

[178] Benda A., Fagul’ová V., Deyneka A., Enderlein J., Hof M. (2006). Fluorescence lifetime correlation spectroscopy combined with lifetime tuning: new perspectives in supported phospholipid bilayer research. Langmuir.

[179] Kułakowska A., Jurkiewicz P., Sỳkora J., Benda A., Mely Y., Hof M. (2010). Fluorescence lifetime tuning—a novel approach to study flip-flop kinetics in supported phospholipid bilayers. J. Fluoresc..

[180] Moerland R. J., Hoogenboom J. P. (2016). Subnanometer-accuracy optical distance ruler based on fluorescence quenching by transparent conductors. Optica.

[181] Gómez-Santos G., Stauber T. (2011). Fluorescence quenching in graphene: a fundamental ruler and evidence for transverse plasmons. Phys. Rev. B.

[182] Gaudreau L., Tielrooij K., Prawiroatmodjo G., Osmond J., de Abajo F. G., Koppens F. (2013). Universal distance-scaling of nonradiative energy transfer to graphene. Nano Lett..

[183] Ghosh A., Sharma A., Chizhik A. I. (2019). Graphene-based metal-induced energy transfer for sub-nanometre optical localization. Nat. Photonics.

[184] Füllbrunn N., Li Z., Jorde L. (2021). Nanoscopic anatomy of dynamic multi-protein complexes at membranes resolved by graphene-induced energy transfer. Elife.

[185] Kamińska I., Bohlen J., Yaadav R. (2021). Graphene energy transfer for single-molecule biophysics, biosensing, and super-resolution microscopy. *Adv. Mater.*.

[186] Ghosh A., Chizhik A. I., Karedla N., Enderlein J. (2021). Graphene-and metal-induced energy transfer for single-molecule imaging and live-cell nanoscopy with (sub)-nanometer axial resolution. *Nat. Protoc.*.

[187] Thiele J. C., Helmerich D. A., Oleksiievets N. (2020). Confocal fluorescence-lifetime single-molecule localization microscopy. ACS Nano.

[188] Ulku A. C., Ardelean A., Antolovic I. M. (2020). Wide-field time-gated SPAD imager for phasor-based FLIM applications. Methods Appl. Fluoresc..

[189] Prokazov Y., Turbin E., Weber A., Hartig R., Zuschratter W. (2014). Position sensitive detector for fluorescence lifetime imaging. J. Instrum..

[190] Michalet X., Siegmund O. H. W., Vallerga J. V., Jelinsky P., Millaud J. E., Weiss S. (2006). Photon-counting H33D detector for biological fluorescence imaging. Nucl. Instrum. Methods Phys. Res., Sect. A.

[191] Bowman A. J., Klopfer B. B., Juffmann T., Kasevich M. A. (2019). Electro-optic imaging enables efficient wide-field fluorescence lifetime microscopy. Nat. Commun..

[192] Sparks H., Görlitz F., Kelly D. J. (2017). Characterisation of new gated optical image intensifiers for fluorescence lifetime imaging. Rev. Sci. Instrum..

[193] Oleksiievets N., Thiele J. C., Weber A. (2020). Wide-field fluorescence lifetime imaging of single molecules. J. Phys. Chem..

[194] Hamans R. F., Parente M., Baldi A. (2021). Super-resolution mapping of a chemical reaction driven by plasmonic near-fields. Nano Lett..

[195] Milagres de Oliveira T., Albrecht W., González-Rubio G. (2020). 3D characterization and plasmon mapping of gold nanorods welded by femtosecond laser irradiation. ACS Nano.

[196] Bruschini C., Homulle H., Antolovic I. M., Burri S., Charbon E. (2019). Single-photon avalanche diode imagers in biophotonics: review and outlook. Light Sci. Appl..

[197] Hirvonen L. M., Suhling K. (2020). Fast timing techniques in FLIM applications. AIP Conf. Proc..

[198] Cuccato A., Antonioli S., Crotti M. (2013). Complete and compact 32-channel system for time-correlated single-photon counting measurements. IEEE Photonics J..

